# Evaluation of lipid coverage and high spatial resolution MALDI-imaging capabilities of oversampling combined with laser post-ionisation

**DOI:** 10.1007/s00216-019-02290-3

**Published:** 2019-12-26

**Authors:** Andrew P. Bowman, Jeroen F. J. Bogie, Jerome J. A. Hendriks, Mansour Haidar, Mikhail Belov, Ron M. A. Heeren, Shane R. Ellis

**Affiliations:** 1 Maastricht MultiModal Molecular Imaging (M4I) Institute, Division of Imaging Mass Spectrometry, Maastricht University, Universiteitssingel 50, 6629 ER Maastricht, The Netherlands; 2 Department of Immunology and Biochemistry, Biomedical Research Institute, Hasselt University, 3590 Diepenbeek, Belgium; 3 Spectroglyph LLC, Kennewick, WA 99338 USA

**Keywords:** Mass spectrometry imaging, MALDI, Lipids, Kidney, Brain, Multiple sclerosis

## Abstract

Matrix-assisted laser desorption/ionisation-mass spectrometry imaging (MALDI-MSI) is a powerful technique for visualising the spatial locations of lipids in biological tissues. However, a major challenge in interpreting the biological significance of local lipid compositions and distributions detected using MALDI-MSI is the difficulty in associating spectra with cellular lipid metabolism within the tissue. By-and-large this is due to the typically limited spatial resolution of MALDI-MSI (30–100 μm) meaning individual spectra represent the average spectrum acquired from multiple adjacent cells, each potentially possessing a unique lipid composition and biological function. The use of oversampling is one promising approach to decrease the sampling area and improve the spatial resolution in MALDI-MSI, but it can suffer from a dramatically decreased sensitivity. In this work we overcome these challenges through the coupling of oversampling MALDI-MSI with laser post-ionisation (MALDI-2). We demonstrate the ability to acquire rich lipid spectra from pixels as small as 6 μm, equivalent to or smaller than the size of typical mammalian cells. Coupled with an approach for automated lipid identification, it is shown that MALDI-2 combined with oversampling at 6 μm pixel size can detect up to three times more lipids and many more lipid classes than even conventional MALDI at 20 μm resolution in the positive-ion mode. Applying this to mouse kidney and human brain tissue containing active multiple sclerosis lesions, where 74 and 147 unique lipids are identified, respectively, the localisation of lipid signals to individual tubuli within the kidney and lipid droplets with lesion-specific macrophages is demonstrated.

**Electronic supplementary material:**

The online version of this article (10.1007/s00216-019-02290-3) contains supplementary material, which is available to authorized users.

**Figure:**
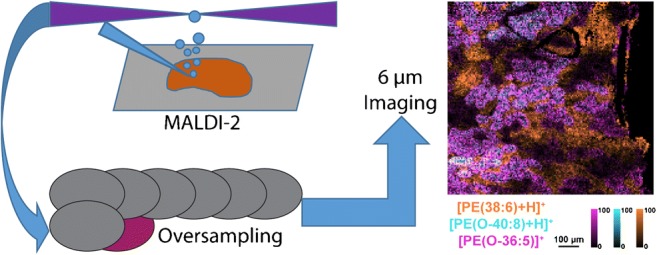
Graphical abstract

## Introduction

Matrix-assisted laser desorption/ionisation-mass spectrometry imaging (MALDI-MSI) is a powerful method for visualising the spatial distributions of lipids throughout biological tissues [1–3]. Its versatility for mapping changing lipid compositions within tissues has been demonstrated in a variety of applications, including oncology [4–6], bacterial infections [7, 8] and liver disease [9, 10], amongst many others. The heterogeneous lipid compositions observed with MSI are ultimately the result of cellular-level lipid metabolism occurring within the cells constituting the tissue sample. However, to date, detailed interpretation of lipid MSI data in terms of cellular-level lipid metabolism has been a major challenge, due to limitations in both technology and informatics. One of the most significant limitations has been the relatively low spatial resolution of conventional methods. Typical experiments are performed at pixel sizes of ~ 30–100 μm, significantly larger than most mammalian cells. As a result, each pixel (spectrum) represents the averaged lipid profile acquired from multiple adjacent cells, each potentially possessing distinct metabolic hallmarks and biological functions. This precludes measuring a lipid profile that is reflective of any individual cell, or even single cell type, within the tissue.

The main limitation of pixel size in MALDI-MSI is the area of desorption/ionisation on the tissue surface, that, in turn, is dependent on the laser spot size. Several groups have reported optical modifications to commercial MALDI-MSI ion sources that have reduced the laser spot size and, thus, achievable pixel sizes, down to < 10 μm and at best ~ 1 μm [11–15]. For example, Kompauer et al. recently reported a modified atmospheric pressure MALDI-MSI source capable of reaching 1.4 μm pixel size and imaging lipids within single cells [12]. A drawback of these approaches for reducing laser spot size is the requirement of modifications to instrument optics and the relatively short depth of focus of narrowly focussed lasers. The latter issue can potentially render the approach sensitive to sample topology or imperfect flatness of the sample stage, which can lead to changing laser fluence and ionisation efficiency across the sample surface. Although recent developments in autofocussing methods to compensate for sample topography can help ameliorate these effects, these are not yet widely used [16].

An alternative method to improve spatial resolution of MALDI-MSI is through the oversampling approach [17]. In oversampling, once all material at given sampling position has been desorbed/ablated the sample stage is moved by a distance smaller than the laser spot size. As a result, only part of the laser beam is used for desorption/ionisation and pixel sizes smaller than the spot size can be achieved. The primary advantage of oversampling is it can be employed on most commercial MALDI systems without hardware modifications, so long as the stage is capable of performing sufficiently small steps. Examples of MALDI-MSI in the oversampling mode include imaging of glycosphingolipids in spleen tissue from a Gaucher disease model at a pixel size of 15 μm [18], phospholipids and sulfatides in brain and lung tissue down to 10 μm pixel size [19] and human colon tissue at a pixel size of ~ 5 μm [20]. High-throughput imaging using continuous raster-mode acquisitions can also generate ions under oversampling-like conditions, where only the edge of the laser beam is generating signal [21], although it has been suggested that severe oversampling conditions can lead to reduced sensitivity in raster-mode MSI [22]. In addition to MALDI, oversampling has also been utilised in IR-MALDESI experiments where a 10-μm pixel size was achieved for cholesterol imaging from human cervical tissue, although the authors noted that at such pixel sizes a dramatic decrease in sensitivity and lipid coverage was observed [23].

A challenge with oversampling using conventional MALDI lasers with Gaussian-like intensity profiles is that only the edges of the laser spot, where the fluence is lowest, is available for desorption/ionisation. This can lead to conditions where, although matrix and analyte are desorbed from the surface, analyte molecules are not efficiently ionised. Such effects can be particularly significant in the conventional pixel-by-pixel acquisition mode [24]. An innovative option to overcome this decreased ionisation efficiency is through the use of laser post-ionisation combined with MALDI-MSI (so-called MALDI-2). MALDI-2 has been demonstrated to enable an up to two order of magnitude increase in sensitivity for lipids and other molecular classes from biological tissues [25–27]. Recently, MALDI-2 has been combined with transmission mode MSI to enable spatial resolutions as low as 600 nm under oversampling conditions [28], but it has not yet been evaluated for oversampling using conventional front-side MALDI laser introduction.

In this work we evaluate for the first time the combination of oversampling MALDI-MSI employing conventional front-side laser introduction with MALDI-2 for the imaging of lipids on a high-resolution Orbitrap mass spectrometer. Exploiting the increased ionisation efficiency enabled by MALDI-2, we demonstrate the ability to generate rich lipid signals from pixel sizes as low as 6 μm from an original laser spot size of ~ 15 μm. In addition, using an automated lipid identification workflow, we have studied the types and numbers of lipid species that can be detected using MALDI and MALDI-2 in both conventional and oversampling imaging modes. This provides what is to date the most comprehensive overview of lipid detection using MALDI-2. The utility of this method for high content and high spatial resolution lipid imaging using 6 μm pixel sizes is demonstrated using rat liver, mouse kidney and human brain tissue containing active multiple sclerosis lesions, where localisation of lipid signal to individual cellular-level features is found. This unique combination of high mass accuracy, high mass resolving power, high spatial resolution and enhanced sensitivity provides an exciting method to study lipid metabolism at the cellular level within heterogeneous and complex tissue sections.

## Methods

### Materials

Isopropanol (LC-MS grade), ethanol (LC-MS grade), 2,5-dihydroxybenzoic acid (DHB, ≥ 99.9% purity), 2,5-dihydroxyacetophenone (DHA) and water (LC-MS grade) were purchased from Sigma Aldrich (Zwijndrecht, The Netherlands) and used without further purification. Haematoxylin (Merck, Darmstadt, Germany) and eosin Y (J.T. Baker, Center Valley, PA, USA) were used under standard laboratory protocols. Indium tin oxide (ITO)-coated glass slides were purchased from Delta Technologies (Loveland, USA).

### Biological samples

Healthy rat liver was obtained from Maastricht University in accordance with protocols approved by the Animal Care and Use Committee (DEC number 2014-120) and was from the same animal used in a recent study [29]. Rats were provided ad libitum access to water and regular chow. One mouse kidney was collected from an LDL R−/− mice from a protocol approved by the Animal Care and Use Committee (DEC number 2014-069) at Maastricht University. Mice were provided ad libitum access water and regular chow. Animals were housed and cared for at the Central Animal Facility of Maastricht University according to local standards. 12-μm-thick sections were prepared using a cryo-microtome (Leica, Nussloch, Germany) at – 20 °C and thaw-mounted on ITO-coated glass slides. Sections were stored at − 80 °C until analysis which occurred approximately 6 months after sectioning. Human brain tissue with active multiple sclerosis (MS) lesions were obtained from the Netherlands Brain Bank (NBB, Amsterdam, The Netherlands). The Netherlands Brain Bank received permission to perform autopsies for the use of tissue and for access to medical records for research purposes from the Ethical Committee of the VU University Medical Center, Amsterdam, The Netherlands. The experiment protocols and methods used for analysing active MS lesions were conducted with the approval of the Netherlands Brain Bank and the Medical Ethical Committee Hasselt University and carried out according to institutional guidelines. Brain tissue was stored at − 80 °C until sectioning; 10-μm-thick sections were prepared using a cryo-microtome and were thaw-mounted onto standard glass slides, transported to Maastricht University on dry ice and then again stored at − 80 °C until matrix application and MSI analysis. The time between sectioning and analysis was less than 1 month.

### Sample preparation

Matrix application was performed via sublimation [30]. Sublimation was performed under the following conditions: 40 mg of DHB dissolved in isopropanol and sublimed for 4 min at 160 °C at a pressure of < 4 × 10^−5^ bar. Samples that were coated in DHA followed slightly different conditions: 40 mg of DHA dissolved in acetone and sublimed for 4 min at 140 °C at a pressure of < 4 × 10^−5^ bar. Samples were then recrystallised in a lab-made apparatus containing 1 mL of 0.5% ethanol in water, at 50 °C for 90 s.

Haematoxylin and eosin (H&E) staining was performed on kidney sections after MALDI imaging. Matrix-coated tissue sections were cleaned of remaining MALDI matrix by immersion in 100% ethanol for 20 s. A standard H&E protocol was then used (95% EtOH, 70% EtOH, H_2_O for 30 s each; haematoxylin for 3 min; H_2_O, 70% EtOH, 95% EtOH each for 30 s; eosin for 1 min; 95% and 100% EtOH for 30 s each; xylene for 2 min). High-resolution optical images of stained tissues were generated using a Mirax Desk scanner (3DHistech, Budapest, Hungary). Unfixed cryosections of human brain tissue were stained with 0.3% Oil Red O (ORO, Sigma) for 10 min to visualise neutral lipids (cholesterol esters). Counterstaining of cell nuclei was done using haematoxylin incubation. Analysis was carried out using a Leica DM 2000 LED microscope and ImageJ software.

CD68 immunostaining and analysis was performed on fixed cryosections of human brain tissue using the antibody anti-CD68 (1:100, cat. #14-0688, Invitrogen) and a species compatible Alexa647 secondary antibody (Life Technologies, A21247) as described previously [31]. To label the myelinated areas, the immune-stained sections were subsequently incubated for 30 min at RT with 2 μM Bodipy® 493/503 solution (ThermoFisher Scientific, D3922) diluted in PBS. Analysis was then carried out using a Nikon eclipse 80i microscope.

### Mass spectrometry instrumentation

All MSI experiments were performed on an Orbitrap Elite mass spectrometer (Thermo Fisher Scientific GmbH, Bremen, Germany) coupled to a reduced pressure ESI/MALDI ion source (Spectroglyph LLC, Kennewick, WA, USA). Further details on the ion source can be found in [32]. The 349-nm MALDI laser (Spectra Physics, Mountain View, CA, USA) was operated at a repetition rate of 100 Hz and pulse energy of ~ 1.0 μJ. The laser was focussed to a spot size of ~ 15 × 12 μm as determined by the size of ablation craters in a matrix layer (Electronic supplementary material (ESM) Fig. S1). Laser post-ionisation (MALDI-2) was performed as previously described for the same experimental setup [26]. Briefly, post-ionisation was achieved using a wavelength tuneable optical parametric oscillator laser system (Ekspla NT-230, Vilnius, Lithuania). The MALDI-2 laser was operated at 260 nm with a post-attenuation pulse energy of 500 μJ. Using adjustable mirrors and a right-angled prism the beam was guided to be parallel with and ~ 250–400 μm above the sample surface where it intersected the desorbed plume generated by the MALDI laser pulse. OPO laser emission was synchronised with the MALDI laser using a digital pulse/delay generator (DG645, Stanford Research Systems, Sunnyvale, USA). Emission from the OPO laser occurred 20 μs after each MALDI laser pulse. The mass spectrometer was operated in positive-ion mode using an ion injection time of 250 ms, automatic gain control (AGC) turned off and a mass range of 350–2000.

### Data acquisition

#### Line scans of rat liver

Rat liver tissue was used for comparison of MALDI and MALDI-2 data generated at different step sizes. Three sets of alternate MALDI and MALDI-2 line scans were acquired in a single experiment (i.e. a line of MALDI data followed by a line of MALDI-2 data) and performed at 20, 15, 10, 8, 6 and 4 μm step sizes for both DHB- and DHA-coated tissues. As the first row in each acquisition does not result in oversampling in the vertical direction, one extra row was acquired at the start of each acquisition but not used for data analysis. This ensured each line scan was acquired under representative oversampling conditions. Line scans were performed using a mass resolution setting of 240,000 (FWHM @ *m*/*z* 400) giving a total scan time of 1.06 s/scan.

#### Imaging of mouse kidney and human brain tissue

Human brain tissue containing active multiple sclerosis lesions and mouse kidney tissues were coated in DHB matrix and analysed using MALDI-2-MSI at a step size of 6 μm and a mass resolution of 120,000 (FWHM @ *m*/z 400), giving a total scan time of 0.67 s/scan. As with the line scans, the first row of each dataset was removed so that all rows were acquired under the same oversampling conditions. To support the identifications of several unexpected compounds, ion trap MS/MS spectra were acquired from one human brain and one kidney tissue section using the DDA-imaging method [33].

### Data analysis and lipid identification

All data image visualisation and data analysis was performed using LipostarMSI (Molecular Horizon Srl, Bettona, Italy). Prior to import, all proprietary Thermo Fisher .raw data was converted into imzML [34]. This was done by first converting raw data into mzML using msconvert (ProteoWizard) [35]. Using the in-built converter of LipostarMSI, the mzmL file was then combined with the positioning file created by the MALDI/ESI injector to generate a profile mode imzML file. Lipid identification within LipostarMSI was performed with reference to the LIPIDMAPS database (.sdf format) [36] and was based on accurate *m*/*z* matching using a tolerance of ± 2 ppm. All lipid identifications are therefore reported to the sum-composition level. Phospholipids, sphingolipids and sterols were considered for identification. Note that in the case of sterols, many isomeric species are possible and we thus group all *m*/z matches to a general “sterol” group, while for ether phospholipids, identifications containing isomeric acyl and alkenyl linkages were grouped into a general ether sub-group (e.g. PE–O and PC–O). In the case of MALDI analysis of liver tissue, potassiated species dominated the spectra, while MALDI-2 spectra were dominated by protonated species, consistent with prior observations comparing MALDI and MALDI-2 [25, 26]. Therefore, to avoid the occurrence of the same lipid being detected as multiple adducts and counting as multiple identifications, only [M+K]^+^ ions of phospholipids and sphingolipids were considered for MALDI data, and only [M+H]^+^ ions considered for MALDI-2 data. Sterols were searched for as [M+H-H_2_O]^+^ ions for both MALDI and MALDI-2, apart from cholesterol esters (CE) for the analysis of human brain tissue (see below). Several sub-classes unlikely to be observed in positive-ion mode data (e.g. sulfatides and cardiolipins) were removed from the search list and only identifications corresponding to even-number acyl/ether chains were considered to limit false positives. Further sample-specific parameters are provided below.

#### Line scans of rat liver

Each line scan was converted to imzML file consisting of 75 pixels (spectra) using a dummy .xml position file. This resulted in three MALDI and three MALDI-2 datasets per step size and matrix type. While no lower limit was set for peak intensity for peak picking during data import, the minimum peak frequency was set to 50% (meaning that peaks had to appear in at least half of the pixels in any given line) with a tolerance for peak alignment of 3 ppm. In this way, only reproducible signals were considered and very low abundance peaks close to the detection limit and/or corresponding to random electronic noise were discarded. All scans were recalibrated using up to three peaks, [cholesterol-H_2_O+H]^+^, [PE(38:4)+H]^+^, and [PC(34:2)+K]^+^, during data import. The final ID list was then manually curated and several seemingly spurious identifications were removed. Single-scan noise values were taken from Xcalibur Qual Browser 2.3 (Thermo Fisher Scientific GmbH, Bremen, Germany).

#### Imaging of mouse kidney and human brain tissue

Import of imzML files to LipostarMSI for the kidney and brain samples was performed using the following parameters: intensity threshold of 1% of base peak, peak alignment tolerance of 3 ppm, peak detection frequency of 2% and a minimum spatial chaos value of 0.7 (with a value of 1 corresponding to high image structure and a value of 0 to a random (structureless) spatial distribution). Identification followed an identical process outlined above, apart from [M+K]^+^ ions of CEs also being considered in the brain data. These were added following manual interrogation of the raw data and the observation of several localised and abundant potassiated CE signals. In addition to manual curation of identified lipid species as outlined for liver line scans above, we also only kept identifications that displayed tissue-specific distributions. Several antioxidant species observed as radical cations from kidney tissue were manually annotated but not counted in the final identification lists as these identifications were not automated (LipostarMSI does not yet support radical cations as a search class).

MSI data was visualised following total ion current normalisation and applying hotspot removal (high quantile 99%).

### Scanning electron microscopy

Scanning electron microscopy images of matrix-coated kidney tissue after DHB sublimation and recrystallisation were acquired using a Philips XL30 microscope.

## Results and discussion

### Lipid coverage with oversampling coupled with MALDI-2 from liver tissue

**Fig. 1:**
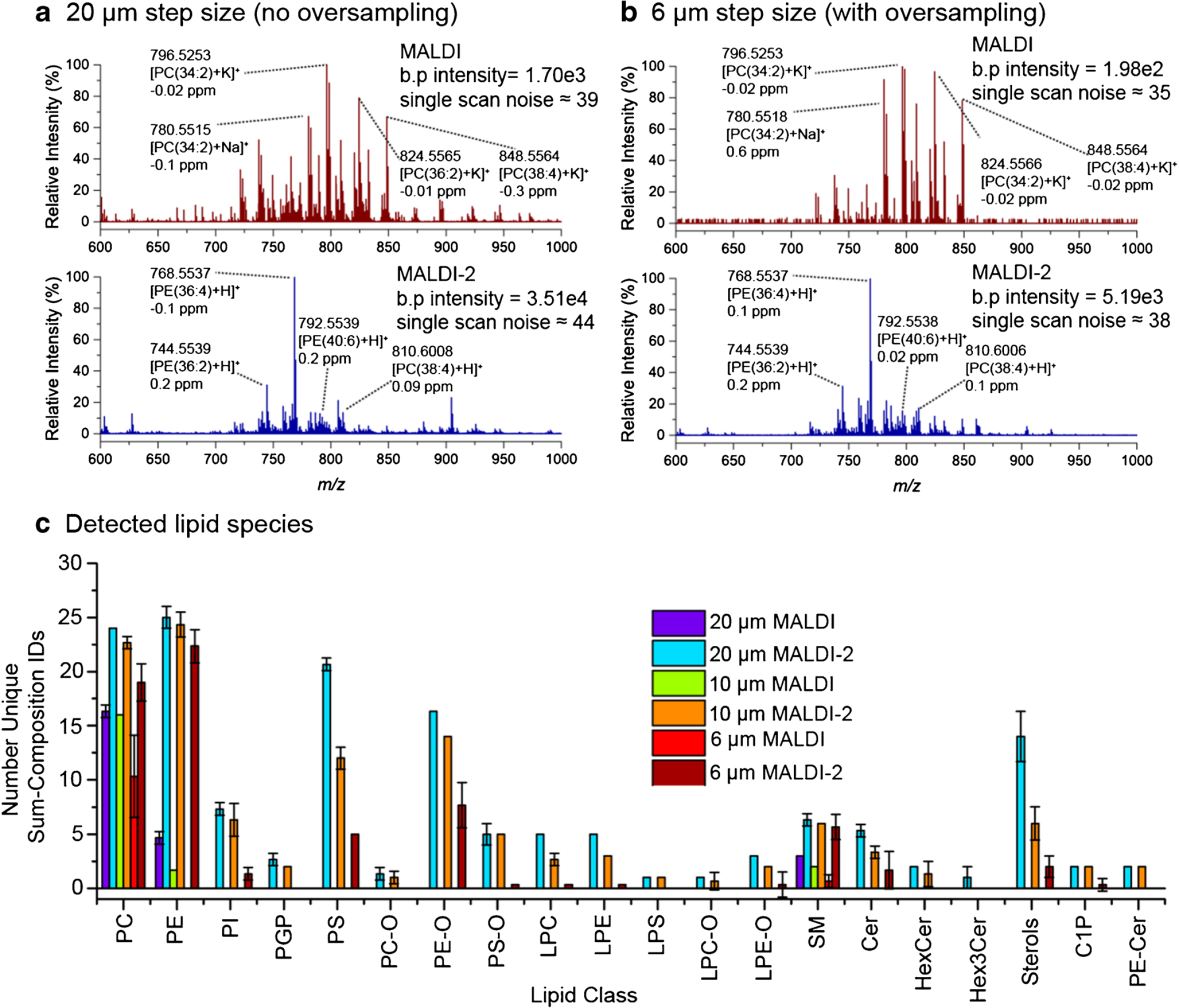
MALDI (top, red trace) and MALDI-2 (bottom, blue trace) spectra acquired from rat liver tissue coated in DHB matrix using stage step sizes of **a** 20 μm (no oversampling) and **b** 6 μm (with oversampling). Each spectrum is the average of 10 consecutive scans. **c** The number of detected lipid species across different lipid classes observed using MALDI and MALDI-2 using 20, 10 and 6 μm line scans across liver tissue. Peaks used for identification had a detection frequency ≥ 50% across the line scans consisting of 75 pixels (equivalent to being detected in half or more individual scans). [M+K]^+^ ions were considered for MALDI identification and [M+H]^+^ ions considered for MALDI-2 identifications, with the exception for sterols which were identified in both cases as [M+H-H_2_O]^+^ ions. Error bars represent ± 1 standard deviation across three replicate line scans. b.p. = base peak

Data for both MALDI and MALDI-2 spectra were generated using stage step sizes of 20 μm (no oversampling) and 6 μm (significant oversampling) from line scans of liver tissue to compare the number and types of lipid species detected. The spot size of the MALDI laser on the tissue was ~ 15 μm × 11 μm (ESM Fig. S1). As expected, at smaller step sizes the total ion current and overall signal-to-noise (S/N) decrease for both MALDI and MALDI-2 data (ESM Fig. S2). When using a 4-μm step size, lipid signals were low and unstable; thus, 4-μm data was not explored further. Figure 1 a shows representative MALDI spectra from rat liver tissue coated with DHB matrix using a step size of 20 μm (10 consecutive scans averaged with single-scan S/N values indicated, *m*/z 600–1000 shown). Full mass range spectra can be found in the ESM Figs. S3 and S4. As typically observed, MALDI spectra are dominated by phosphatidylcholine (PC) and sphingomyelin (SM) lipids, with their potassiated adducts being most abundant. By contrast, MALDI-2 spectra generated under analogous conditions (Fig. 1a, bottom) resulted in both a ca. 200-fold increase in base peak intensity and dramatic increases in signal for a variety of lipid species, while lipid-related signals also became dominated by [M+H]^+^ ions, consistent with prior observations [25, 26]. The benefit of MALDI-2 is observed to hold even under oversampling conditions. Figure 1 b (top) shows representative MALDI spectra acquired using a 6-μm step size that results in significant oversampling. While the overall spectrum resembles that shown in the top panel of Fig. 1 a, the signal intensity is reduced by 10-fold with many peaks no longer being detected. However, data acquired using a 6-μm step size and MALDI-2 still produces high S/N spectra with base peak intensity ~ 3-fold higher than those generated by conventional MALDI at 20 μm (Fig. 1b, bottom). Using a 6-μm step size, the S/N of the base peak corresponding to the phosphatidylethanolamine (PE), [PE(36:4)+H]^+^, is 136 using MALDI-2, whereas using the same step size, the corresponding lipid detected with conventional MALDI, observed as the [M+K]^+^ ion, has a single-scan S/N of < 1. For the abundant PC(34:2) lipid, the signal intensity for the protonated species acquired at 6 μm step size was comparable to that measured for the abundant potassiated species with MALDI at 20 μm step size. It is noteworthy for these data acquired at 6 μm step size that individual spectra are taken from a tissue area equivalent to or smaller than the size of a typical mammalian cell (10–100 μm), thereby providing data that is reflective of cellular-level lipid compositions within the tissue, although we acknowledge that for smaller cells there is a reasonable probability of collecting ion signal from two adjacent cells in a single pixel.

MALDI-2 oversampling was also evaluated using 2,5 DHA matrix (ESM Fig. S5). Using a 20-μm step size, MALDI and MALDI-2 spectra were similar to those obtained using DHB. Interestingly though, with an increasing extent of oversampling, many of the MALDI-2-specific signals observed with DHB yielded lower relative intensities using DHA. For example, at 6 μm step sizes using MALDI-2, protonated PC signals yielded the highest signal intensities (rather than PE lipids), while a relative increase in the abundance of potassiated PC lipids compared to their protonated forms was also observed. In contrast, the overall spectral profile at different step sizes remained relatively consistent using DHB (Fig. 1a, b). The origin of this effect is unclear, but appears to suggest a shift of DHA ionisation properties under oversampling conditions when using MALDI-2. We speculate this effect is related to (i) the higher volatility of DHA, resulting in a larger desorption area upon irradiation by the MALDI laser. Upon oversampling, this could result in matrix further from the centre of the laser spot being desorbed leading to a plume of lower density in which MALDI-2 can occur; and/or (ii) possible differences in matrix morphology and diffusion rates of lipids from the tissue with lipids closer to the surface being more selectively desorbed by the edge of the laser beam. Different diffusion rates of lipids have recently been reported in sublimed matrix coatings using secondary ion mass spectrometry [37]. Thorough investigation of this effect lies outside the scope of this work, but provides an exciting avenue to pursue that could provide insight into the MALDI-2 mechanism.

We next evaluated the breadth of lipid coverage that can be obtained in such high spatial resolution MSI experiments using DHB. Peaks used for automated identification were defined during data import as having a peak detection frequency ≥ 50% (equivalent to being detected in half or more scans/pixels within a 3-ppm tolerance) across each 75 pixel line scan. Figure 1 c shows the number of lipid species detected within 2 ppm of their theoretical *m*/*z* for both MALDI and MALDI-2 at step sizes of 20 μm (no oversampling), 10 μm (moderate oversampling) and 6 μm (significant oversampling). The full list of tentatively identified species and their abundances is provided in ESM Table S1. Conventional MALDI detected primarily PC lipids along with several abundant SM and PE lipids as [M+K]^+^ ions. In total, 24, 19 and 11 unique sum-composition lipid species were detected with MALDI at step sizes of 20, 10 and 6 μm, respectively. Dramatically more lipid species were detected using MALDI-2, with 149, 117 and 66 lipid species being detected as [M+H]^+^ ions ([M+H-H_2_O]^+^ for sterols) at 20, 10 and 6 μm step sizes, respectively. While PE and PE–O species were amongst the biggest beneficiaries of MALDI-2, it also enabled the detection of a diverse array of lipid classes not observed with conventional MALDI. For example, MALDI-2 could detect both phosphatidylinositol (PI) and phosphatidylserine (PS) lipid species as [M+H]^+^ ions. These lipids are often only observable in negative-ion mode MALDI analysis thereby demonstrating the added lipid coverage offered by MALDI-2. Supporting these observations is the fact that the most intense protonated species observed for each, PI(38:4) and PS(36:1), have been shown to be the two most abundant species of each class in liver using LC-MS/MS [38]. Despite the expected drop in the number of detected lipids with decreasing pixel size, the above data demonstrates that rich lipid MSI data covering many different lipid species can be generated from pixel sizes as low as 6 μm, with a ~ 3-fold increase in the number of detected lipid species compared to conventional MALDI using a 20-μm pixel size.

### High spatial resolution imaging of lipids in kidney tissue

**Fig. 2:**
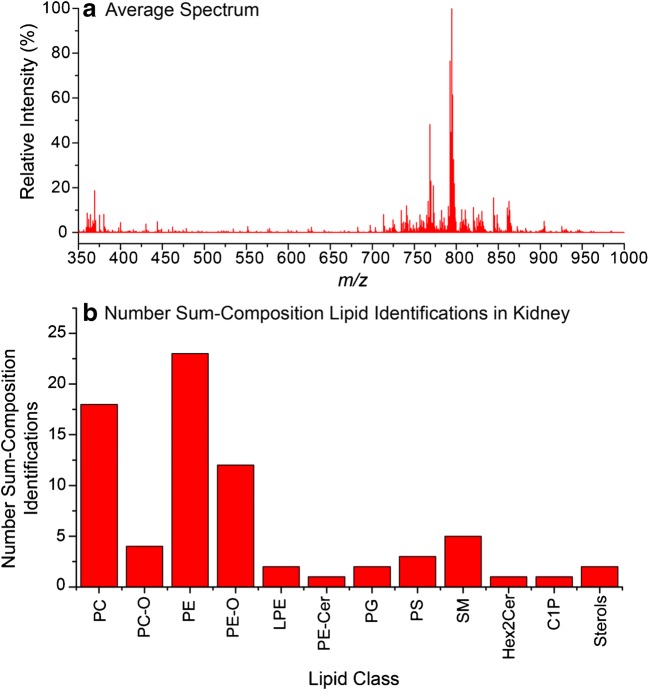
**a** Average spectrum acquired from mouse kidney tissue using MALDI-2 and a pixel size of 6 μm between *m*/*z* 350 and 1000. **b** Number of automatically identified lipid species from mouse kidney tissue. Lipids were identified as [M+H]^+^ ions ([M+H-H_2_O]^+^ for sterols) using an *m*/*z* tolerance of 2 ppm

Mouse kidney tissue was used to evaluate the high-resolution imaging capabilities of MALDI-2 oversampling. A ~ 4-mm^2^ area of DHB-coated tissue section was analysed using a 6-μm step resulting in an image consisting of 108,558 pixels. Using scanning electron microscopy, the DHB crystal sizes were found to be significantly smaller than 6 μm (ESM Fig. S6). The average spectrum is shown in Fig. 2 a and demonstrates the detection of a variety of lipid species across the *m*/*z* 350–2000 mass range. Using the described approach for automated lipid identification (see “Methods”), 74 unique sum-composition lipid species were identified as [M+H]^+^ ions ([M+H-H_2_O]^+^ ions for sterols) from this dataset. The distribution of these identifications across the different lipid sub-classes is shown in Fig. 2 b. As observed for the liver data above, PC, PE and PE–O lipids constituted the majority of detected lipid species, and in total, 12 different lipid sub-classes could be identified and imaged. The full list of identified lipids is provided in ESM Table S2.

**Fig. 3:**
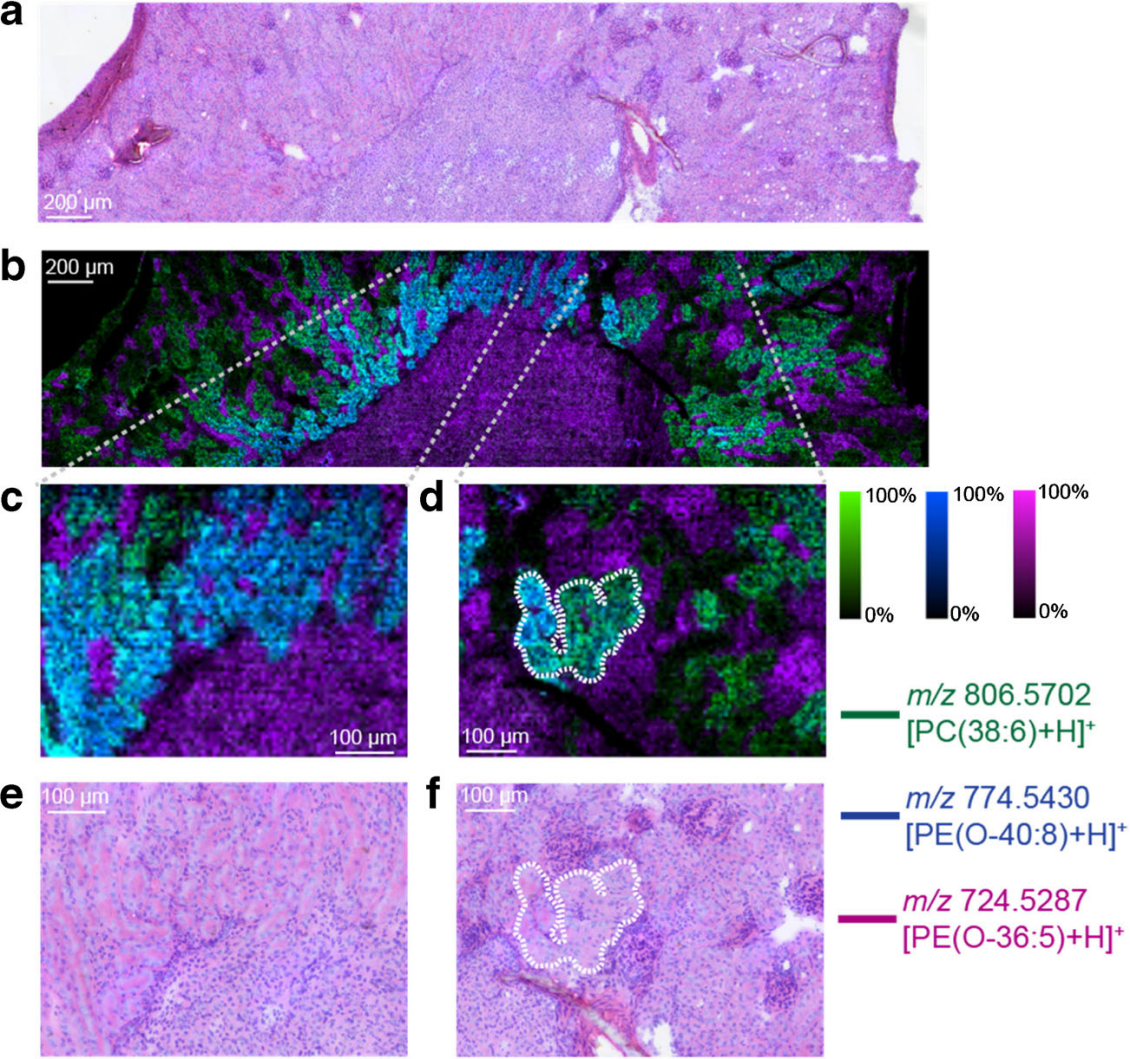
**a** Optical image of the post-MSI H&E-stained tissue section. **b** Ion distribution images of [PC(38:6)+H]^+^ (green), [PE(O-40:8)]^+^ (blue) and [PE(O-36:5)+H]^+^ (pink) throughout mouse kidney tissue acquired using MALDI-2 and a pixel size of 6 μm. **c**, **d** Selected enlarged regions of the MSI data. The corresponding H&E images of these enlarged regions are shown in **e** and **f**. All MSI data is visualised using total ion current normalisation and hotspot removal (99% quantile)

Figure 3 shows an optical image of the post-MSI H&E-stained tissue section (Fig. 3a) and an overlay of three identified lipid species (Fig. 3b, [PC(38:6)+H]^+^ (green), [PE(O-40:8)+H]^+^ (blue) and [PE(O-36:5)+H]^+^ (pink)). Using the distributions of these three ion signals, a number of different tissue regions can be localised at the cellular level. [PE(O-36:5)+H]^+^ is localised to the inner medulla, inner stripe, glomeruli and the interstitium regions of the kidney. Both [PE(O-40:8)+H]^+^ and [PC(38:6)+H]^+^ are specific to the kidney tubuli, with [PC(O-40:8+H]^+^ being more abundant in tubuli contained within the outer stripe of the medulla and [PC(38:6)+H]^+^ being more abundant in tubuli contained within the cortex. The high specificity for these lipid signals for histologically different tissue regions can be seen in the zoomed MSI and H&E data shown in Fig. 3 c–f. The outlined area of Fig. 3 d (white dotted line) highlights tubuli-specific lipid signals corresponding to the tubular regions outlined in the H&E-stained tissue with excellent spatial specificity (Fig. 3f). This strong agreement between the MSI and histological data confirms the high spatial resolution enabled by both the oversampling method and that the employed sample preparation using sublimation minimises analyte delocalisation. Combined, this data demonstrates the ability to achieve pixel sizes on the scale of cellular-level features within tissues while still being able to detect and identify numerous lipid species.

**Fig. 4:**
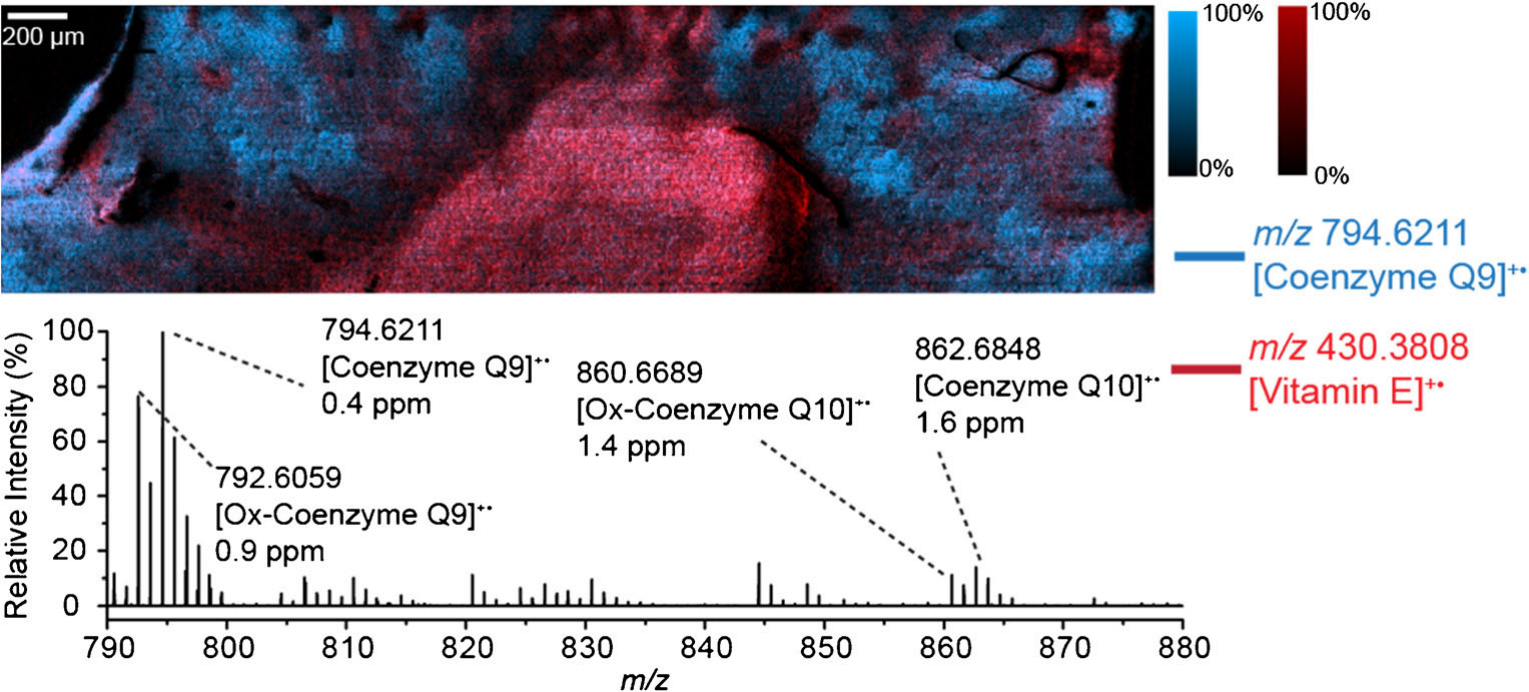
Ion distribution images of *m*/*z* 794.6211 ([coenzyme Q9]^+•^, blue) and *m*/*z* 430.3808 ([vitamin E]^+•^, red) throughout mouse kidney tissue. The corresponding MALDI-2 mass spectrum showing the detection of both the oxidised and reduced forms of coenzyme Q9 and coenzyme Q10 is shown below

In addition to the [M+H]^+^ and [M+H-H_2_O]^+^ ions automatically annotated, we also observed a variety of radical cations corresponding to different lipid-soluble antioxidants within the mouse kidney tissue. These species are generated by direct absorption of the 260-nm MALDI-2 laser light via a [1 + 1] resonance enhanced multiphoton ionisation (REMPI) process. In particular, we observed the radical cation of vitamin E at *m*/*z* 430.3808 which exhibited elevated signal within the inner medulla (Fig. 4, red). We also observed both the oxidised and reduced forms of coenzyme Q9 (*m*/z 792.6059 and *m*/*z* 794.6211) and coenzyme Q10 (*m*/*z* 860.6689 and *m*/*z* 862.6848) as shown in the Fig. 4 spectrum. These constituted abundant signals with the reduced coenzyme Q9 corresponding to the base peak in the spectrum when using a 6-μm step size (Fig. 2a). MS/MS spectra supporting the identification of these radical species are provided in ESM Figs. S7 and S8. The high signals for these species can possibly be explained by a higher efficiency REMPI process that is decoupled from the MALDI-based ionisation processes. All four of these ion signals exhibited similar distributions and were observed throughout most of the tissue with the distribution of the reduced form of coenzyme Q9 shown in blue in Fig. 4. While the protonated forms of oxidised and reduced coenzyme Q9 and Q10 have been detected using MALDI-MSI from brain tissue [39], to our knowledge, this is the first report of their detection in renal tissue with MSI.

### Lipid imaging of human brain tissue containing active multiple sclerosis lesions

Finally, the lipid imaging capabilities of MALDI-2 coupled with oversampling were evaluated using active human multiple sclerosis tissue where the high spatial resolution enabled specific lipid accumulations to be visualised within the tissue lesions. During multiple sclerosis, an autoimmune response is directed against the lipid-rich myelin sheath surrounding axons. Myelin is broken down and cleared by phagocytes which causes failure of axonal conduction, and depending on the affected region, disease symptoms such as impaired muscle control, balance, vision and speech. Myelin processing within the phagocytes leads to the release of lipid mediators that direct the function of the phagocytes and thereby lesion progression and resolution [40–42]. More generally, lipids have been identified to play important roles in multiple sclerosis [43–46], but the precise processes and mechanism by which this occurs, along with the specific functions of individual lipid molecules, remain unknown.

**Fig. 5:**
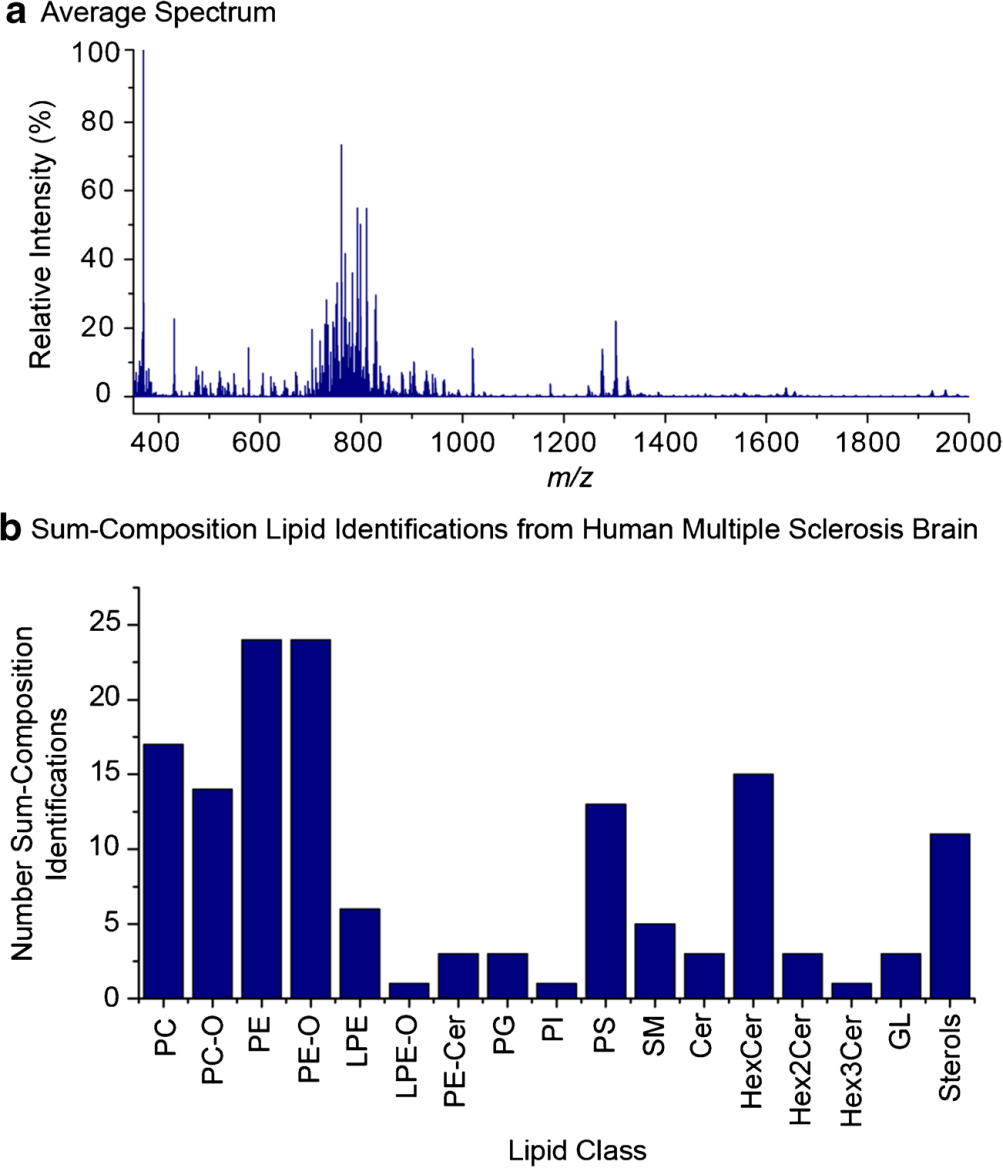
**a** Average spectrum acquired from human multiple sclerosis brain tissue using MALDI-2 and a pixel size of 6 μm between *m*/*z* 350 and 2000. **b** Number of automatically identified lipid species from human multiple sclerosis brain tissue. Lipids were identified as [M+H]^+^ ions ([M+H-H_2_O]^+^ for sterols and [M+K]^+^ for cholesterol esters) using an *m*/*z* tolerance of 2 ppm

Within the imaging dataset acquired from an ~ 4.1-mm^2^ area of tissue and consisting of 114,263 pixels, 147 unique sum-composition lipid species were identified (see “Methods” for identification details). Each of these lipid species revealed tissue-specific distributions throughout the brain tissue. The mean spectrum from this dataset is provided in Fig. 5 a, while the distribution of the lipids across the different classes is shown in Fig. 5 b. Again PC and PE lipids contribute the largest fraction of identified species. Other species with significant contributions to the identified list were sterols, PS and hexosylceramides (HexCer) species. The detection of a number of glycosphingolipids is consistent with their known high abundance and diversity in brain tissue [47]. The full list of identified species is provided in ESM Table S3.

**Fig. 6:**
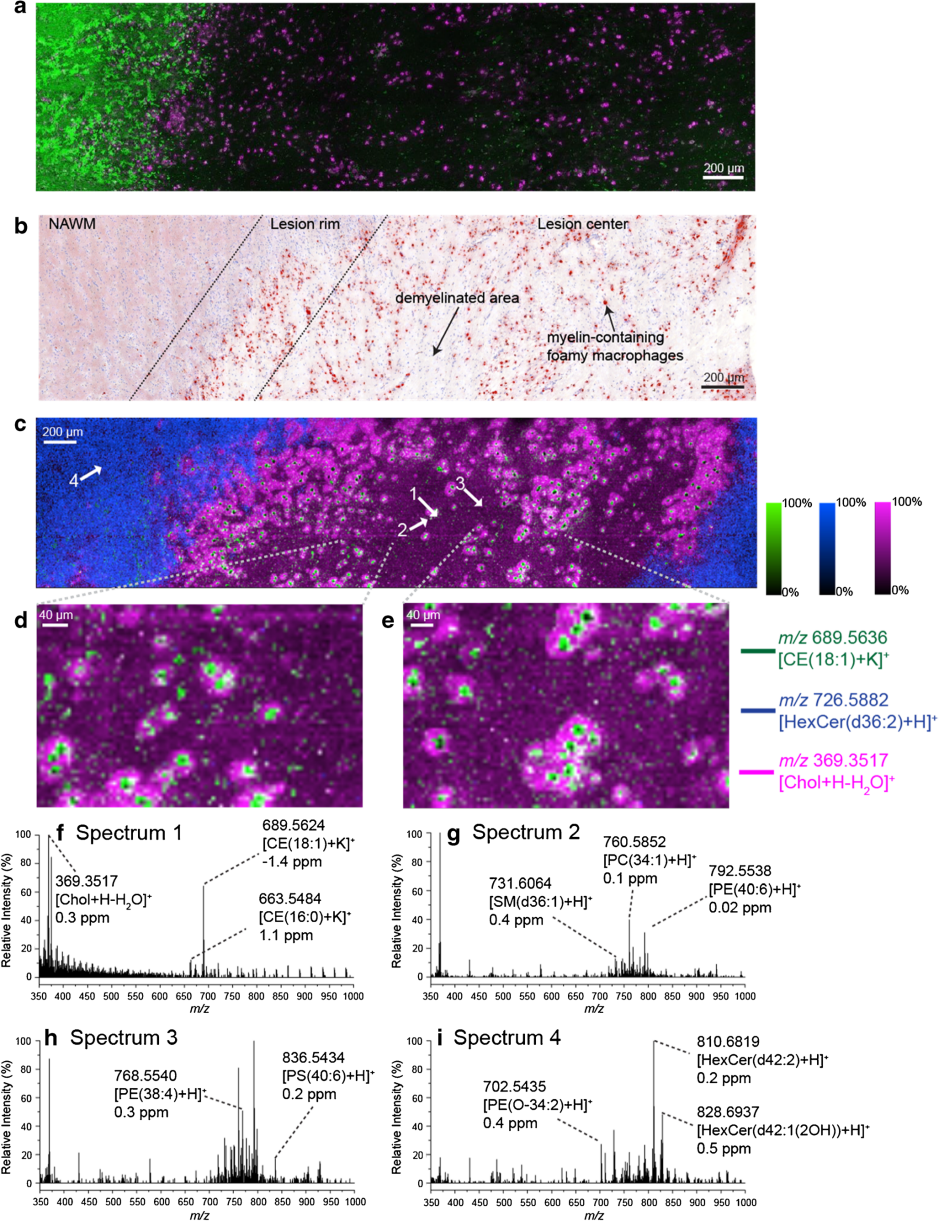
**a** CD68 (macrophages, purple) and Bodipy (myelin/neutral lipids, green) immunostaining and **b** Oil Red O staining of human brain tissue slices acquired from the same patient used to collect the MSI data shown in **c**–**e** with several tissue regions indicated. **c** Ion distributions images of *m*/*z* 689.5636 ([CE(18:1)+K]^+^, green), *m*/*z* 726.5882 ([HexCer(d36:2)+H]^+^, blue) and *m*/*z* 369.3517 ([Chol+H-H_2_O]^+^, pink) acquired using MALDI-2 and a pixel size of 6 μm from human multiple sclerosis brain tissue. **d**, **e** Selected enlarged regions of the MSI data shown in **c**. **f**–**i** Single pixel spectra acquired from the regions indicated by the white arrows in **c**. All MSI data is visualised using the total ion current normalisation and hotspot removal (99% quantile). NAWM = normal appearing white matter

MSI of the human brain tissue using a 6-μm pixel size yielded both high contrast and spatially specific signals for many lipid species, thereby allowing visualisation of fine structural features only 10–20 μm in size. Histopathological analysis was performed on a multiple sclerosis lesion acquired from the same patient revealing this lesion to contain abundant macrophages and microglia (CD68 staining, Fig. 6a). These macrophages show a foamy appearance and are filled with esterified cholesterol and other neutral lipids as demonstrated with a Bodipy (Fig. 6a) and ORO (Fig. 6b) staining. The high single pixel spectral quality and rich lipid signals achieved from only 6 μm pixels is demonstrated in Fig. 6 f-i that show the corresponding spectra obtained from the individual pixels indicated by the white arrows in Fig. 6 c. The overlaid distributions of three lipid ions, [Chol+H-H_2_O]^+^ (pink), [HexCer(d36:2)+H]^+^ (blue) and the cholesterol ester (CE) [CE(16:0)+K]^+^ (green), are shown in Fig. 6 c and clearly correlate with distinct tissue regions. Two enlarged regions of the MSI data are shown in Fig. 6 d and e to further highlight these specific distributions. Analogous distributions were also observed in brain tissue acquired from a second multiple sclerosis patient (ESM Fig. S9). Signal for [HexCer(d36:2)]^+^ was localised to the myelin surrounding the multiple sclerosis lesion and was virtually absent within the lesion. Signal for the CE species [CE(18:1)+K]^+^ is localised in areas only 10–20 μm wide and corresponded to the lipid accumulations within the phagocytes as shown in the ORO staining (Fig. 6a, b). A similar distribution is also observed for the potassium adduct of CE(16:0) (ESM Fig. S10a). Interestingly, the signal for [Chol+H-H_2_O]^+^, likely representing both free cholesterol and in-source fragmentation of CEs, localised to a wider region adjacent to the centres of the phagocyte lipid accumulations. Also, abundant higher *m*/*z* signals observed at *m*/*z* 1302.2069 and *m*/*z* 1276.1906 assigned based on accurate mass to the protonated CE dimer species ([2CE(18:1)+H]^+^ (− 0.3 ppm error) and [CE(18:1)+CE(16:0)+H]^+^ (− 1.0 ppm error) yielded similar distributions as observed for [Chol+H-H_2_O]^+^ (ESM Fig. S10b and S10c). Evidence fo**r** the formation of [2M+H]^+^ ions of CE was obtained upon analysing a CE(18:0) standard using MALDI-2 that yielded an analogous [2CE(18:0)+H]^+^ dimer (ESM Fig. S11). Despite the CE-related signals in the regions around the phagocytes, there is little signal in the ORO staining within these regions. ORO staining is widely thought to be specific for neutral lipid droplets (e.g. those containing CE and triacylglyceride lipids) and does not strongly stain other cellular/tissue regions outside these regions that contain other lipid species. We therefore speculate that the lack of ORO staining in the regions adjacent to the phagocytes is caused by the presence of other lipid species. For example, two PC–O species, ([PC(O-40:7)+H]^+^ and [PC(O-40:6)+H]^+^, were also found specifically in the regions adjacent to the phagocytes (ESM Fig. S10). We also note that the mechanism leading to alkali adducted CE species being observed in the phagocytes and protonated-related signals surrounding these regions is unknown. Nonetheless, these results suggest specific functions of both free cholesterol and CEs in multiple sclerosis and, in particular, phagocyte metabolism. Accumulation of cholesterol in macrophages activates the nuclear liver-X-receptors that modulate cellular lipid metabolism and the production of inflammatory mediators [41]. Interestingly, aging was shown to promote cholesterol accumulation in phagocytes which stimulates the inflammatory activity of these immune cells and impairs their reparative properties [48, 49]. More generally, the localisation of certain lipid species to phagocytes within the brain tissue confirms the high, cellular-level, spatial resolution that can be achieved with this method, while still obtaining rich lipid spectra. This broad lipid coverage and high spatial resolution enabled MALDI-2 coupled with oversampling can provide a new tool to study in-depth the lipid-specific alterations of different cell populations within multiple sclerosis progression as well as other neurodegenerative diseases.

## Conclusions

In this work, we have demonstrated that MALDI-2 combined with an oversampling acquisition approach is capable of both generating rich lipid spectra from tissue and imaging lipids at pixel sizes as low as 6 μm, without modifications to the optics or stage of the commercially available ion source. MALDI-2 data generated from 6 μm pixels was capable of detecting three times more lipid species than conventional MALDI acquired without oversampling at 20 μm pixel size from rat liver tissue. From kidney and brain tissue imaging experiments, we could identify 74 and 147 unique lipid species, in addition to visualising their spatial distributions within the tissue. Importantly, lipid spectra obtained from individual pixels represent an area equivalent to or less than the size of a typical mammalian cell. This is an important capability that will greatly enhance the ability to interpret lipid MSI data in terms of cellular-level lipid metabolism occurring within biological tissues, while preserving the context of the cell within the tissue microenvironment. For example, data acquired from mouse kidney clearly enables the localisation of lipid signals to individual tubuli, while data from human multiple sclerosis tissue enables lipid accumulations within lesion-specific macrophages to be visualised. The combination of information-rich spectra and cellular-level spatial resolutions provides a powerful approach to study spatial and cell-type-specific alterations in lipid metabolism within many different disease types.

From a technology standpoint, this work provides the most comprehensive overview of the lipid detection capabilities of MALDI-2 reported to date, demonstrating the detection of many more lipid classes than possible with positive-ion mode MALDI. Moreover, the coupling of this approach with a new automated lipid identification workflow utilising accurate *m*/*z* measurements enables the rich lipidomics information acquired in such experiments to be readily exploited.

## Electronic supplementary material

ESM 1 (PDF 858 kb)

Table S1 (XLSX 113 kb)

Table S2 (PDF 168 kb)

Table S3 (PDF 246 kb)
