# Supplementary material for: Evaluation of lipid coverage and high spatial resolution MALDI-imaging capabilities of oversampling combined with laser post-ionisation
Source: Anal Bioanal Chem. 2019 Dec 26;412(10):2277–89. doi: 10.1007/s00216-019-02290-3 (PMC7118047; doi:10.1007/s00216-019-02290-3)
Supplement: Supplementary file 1 — (PDF 858 kb) [file 216_2019_2290_MOESM1_ESM.pdf]

Analytical and Bioanalytical Chemistry

Electronic Supplementary Material

Evaluation of lipid coverage and high spatial resolution MALDI-imaging capabilities of oversampling combined with laser post-ionisation

Andrew P. Bowman, Jeroen F. J. Bogie, Jerome J. A. Hendriks, Mansour Haidar, Mikhail Belov, Ron M. A. Heeren, Shane R. Ellis

Additional files available under "Supplementary material".

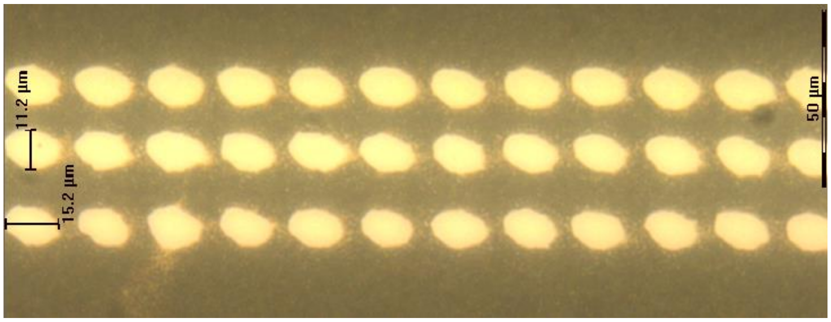

Fig. S1 Optical image of ablation craters generated by the MALDI laser on a DHB-coated slide. MALDI laser spot size is ~11 µm ×15 µm. Laser was operated at 1.1 µJ and a repetition rate of 100 Hz. Stage step size was 20 µm

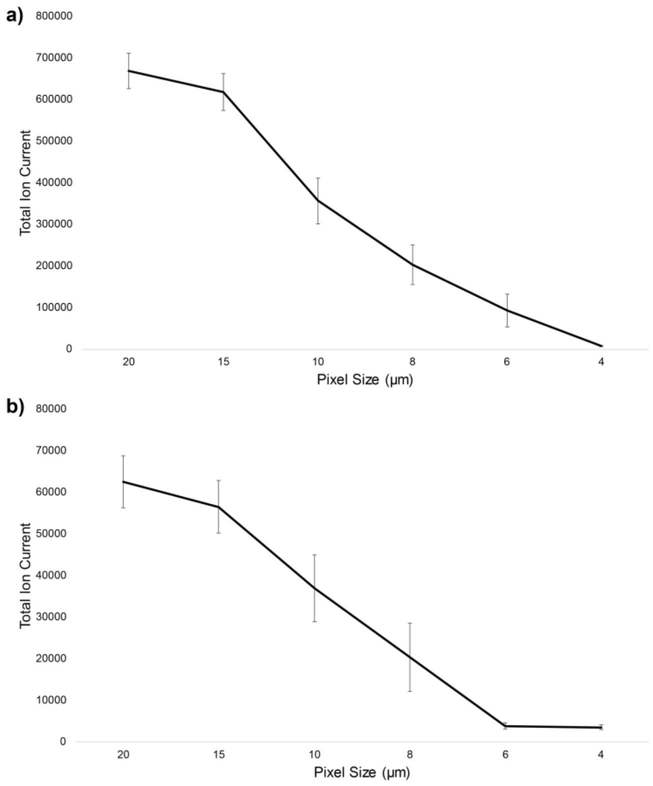

Fig. S2 Total ion current recorded between *m/z* 350-2000 for (a) MALDI-2 and (b) MALDI analysis rat liver tissue at different stage step (pixel) sizes. Error bars represent ± 1 standard deviation of all scans (> 150 individual scans) acquired at a given step size

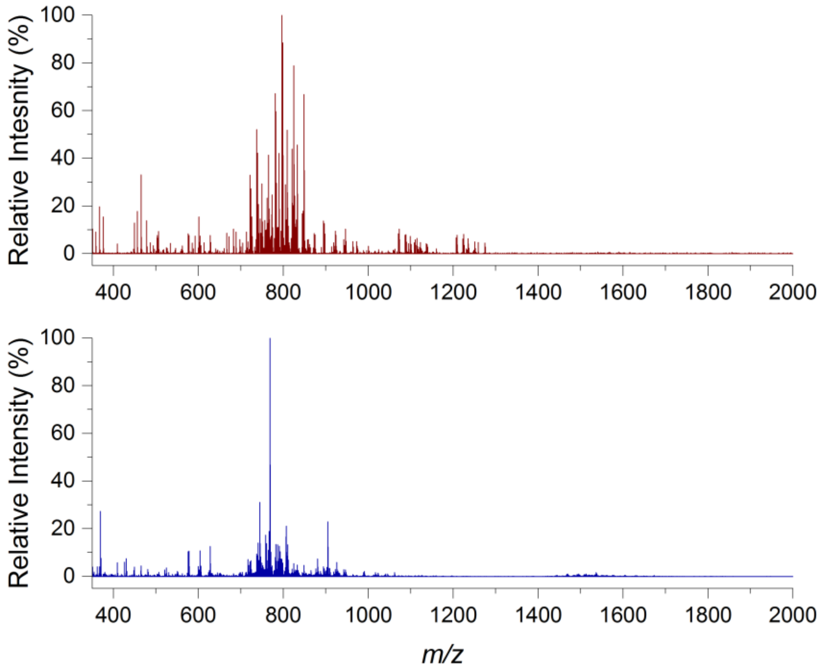

Fig. S3 MALDI (top, red trace) and MALDI-2 (bottom, blue trace) spectra acquired from rat liver tissue using DHB matrix and a 20 µm stage step across the full *m/z* 350-2000 spectral range. Spectra represent the average of 10 consecutive scans

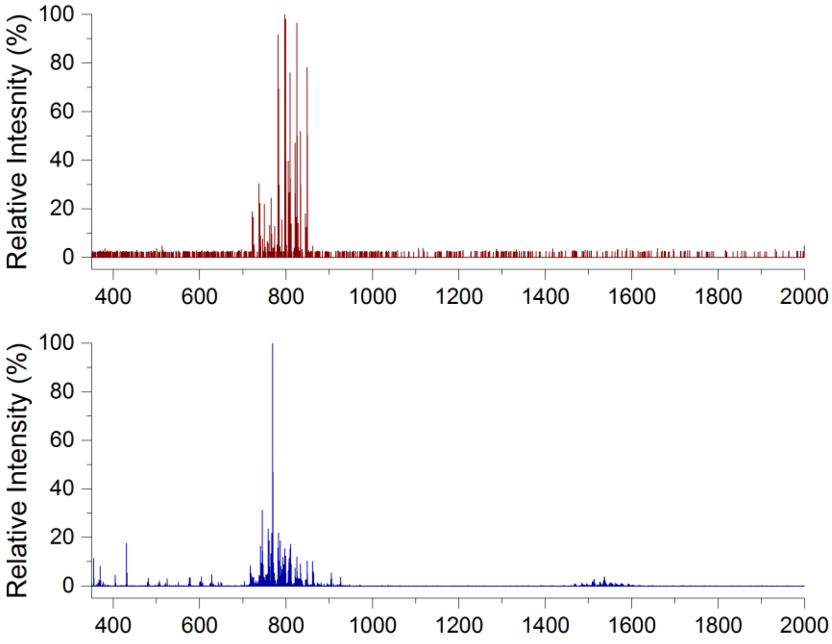

Fig. S4 MALDI (top, red trace) and MALDI-2 (bottom, blue trace) spectra acquired from rat liver tissue using DHB matrix and a 6 µm stage step across the full *m/z* 350-2000 spectral range. Spectra represent the average of 10 consecutive scans

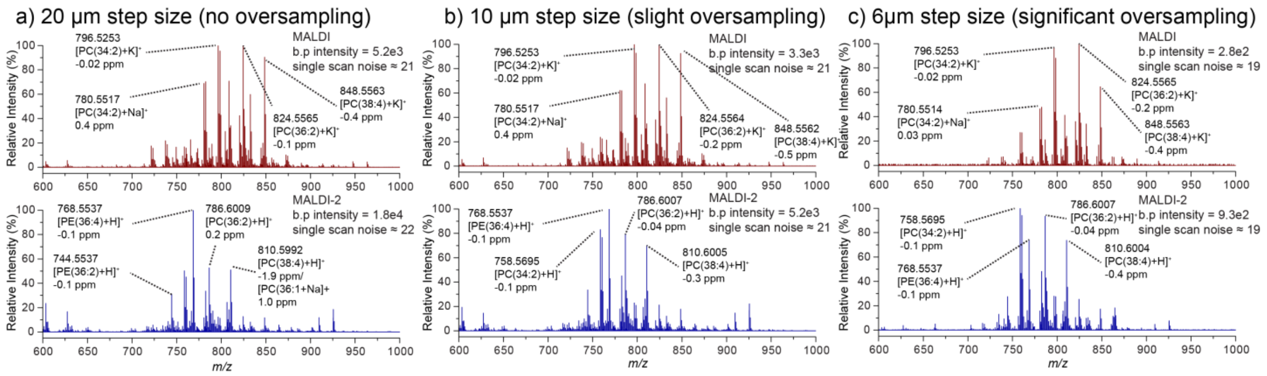

Fig. S5 MALDI (top, red trace) and MALDI-2 (bottom, blue trace) spectra acquired from rat liver tissue using DHA matrix at stage step sizes of 20 µm (left), 10 µm (middle) and 6 µm (right). Spectra represent the average of 10 consecutive scans. The corresponding base peak (b.p) intensity and single scan noise values are indicated

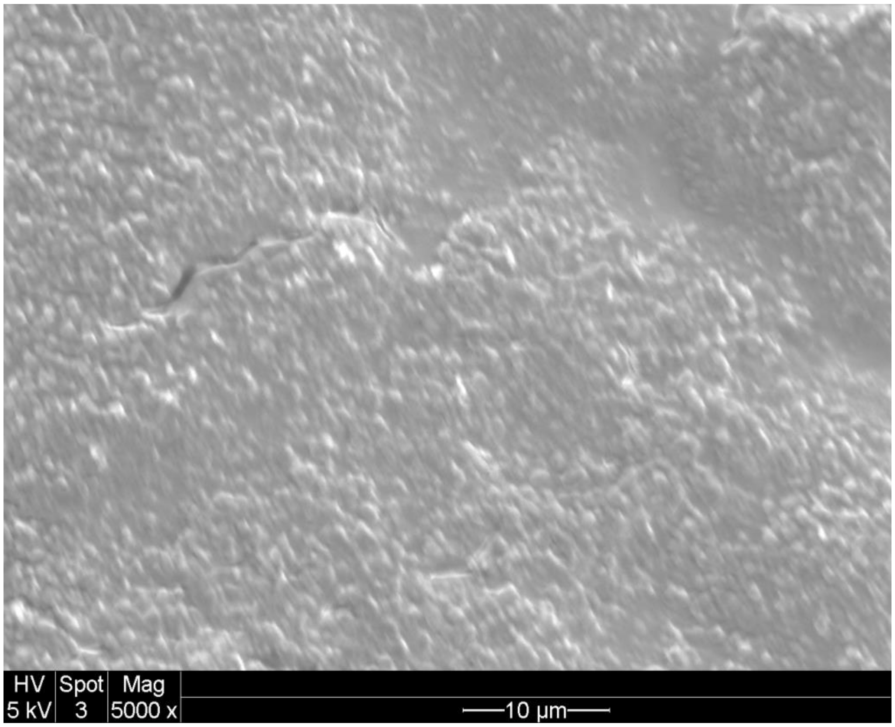

Fig. S6 Scanning electron microscopy images of mouse kidney tissue coated with DHB matrix. DHB matrix was applied using sublimation and then recrystallized as described in the methods section

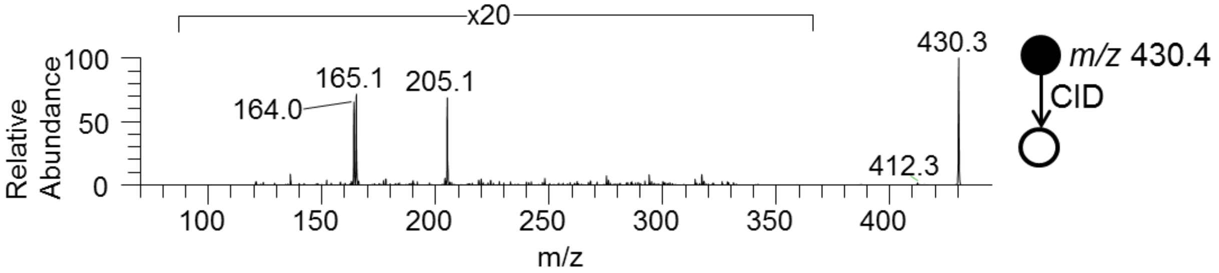

Fig. S7 Ion trap MS/MS spectrum of *m/z* 430.4±0.5 acquired from mouse kidney tissues using MALDI-2. The MS/MS spectrum confirms the identity of the precursor ion at 430.3808 as the radical cation of Vitamin E. Fragments at *m/*z 164.0, 165.1 and 205.1 are consistent with those observed in the electron ionisation spectrum of the same precursor ion [1]

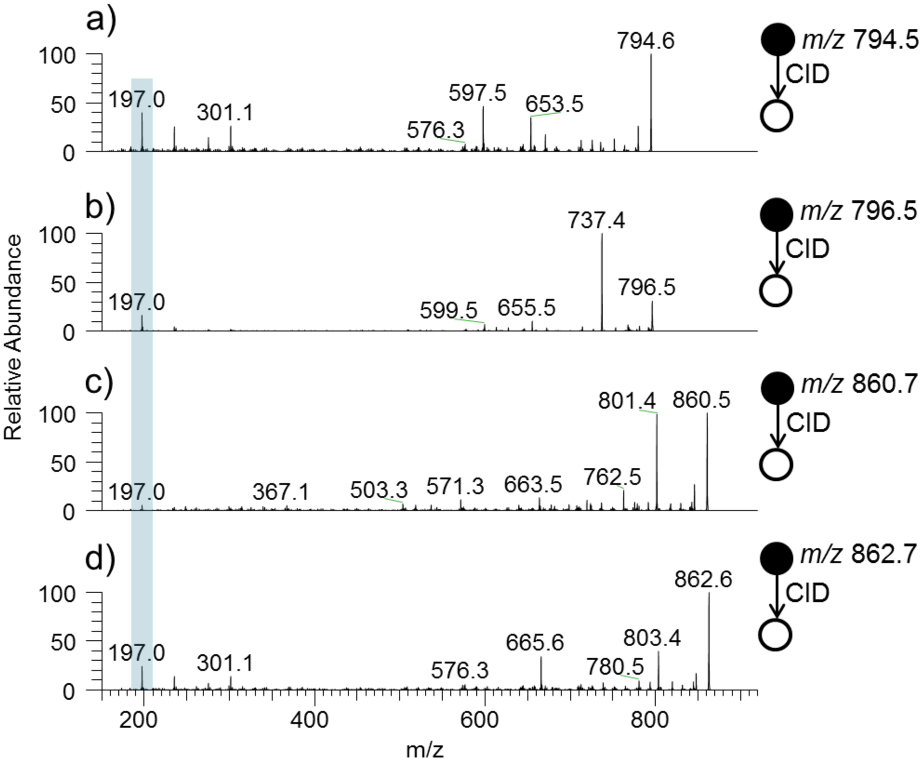

Fig. S8 Ion trap MS/MS spectrum of (a) *m/z* 794.5±0.5, (b) *m/z* 796.5±0.5, (c) *m/z* 860.7±0.5 and (d) *m/z* 862.7±0.5. The MS/MS spectra supports the identification of the precursor ions observed at *m/z* 792.6059, *m/z* 796.6211, *m/z* 860.6689 and *m/z* 862.6848 in Figure 4 as oxidised coenzyme Q9, coenzyme Q9, oxidized coenzyme Q10 and coenzyme Q10, respectively. The detection of a common radical fragment ion is consistent with that reported in the literature [2]

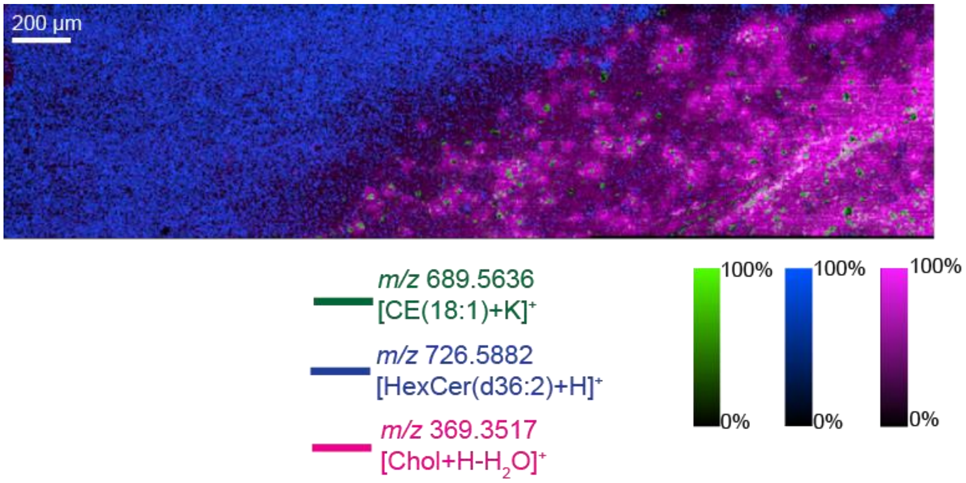

Fig. S9 MALDI-2 MSI data of human brain tissue contacting active multiple sclerosis lesions that was obtained from a different patient compared to that shown in Figure 6. Data was acquired using a 6 µm step size and shows the distribution of *m/z* 689.5636 ([CE(18:1)+K]$^+$, green), *m/z* 726.5682 ([HexCer(d36:2)+H]$^+$, blue) and [Chol+H-H_2O]$^+$, pink). Scale bar is 200 µm

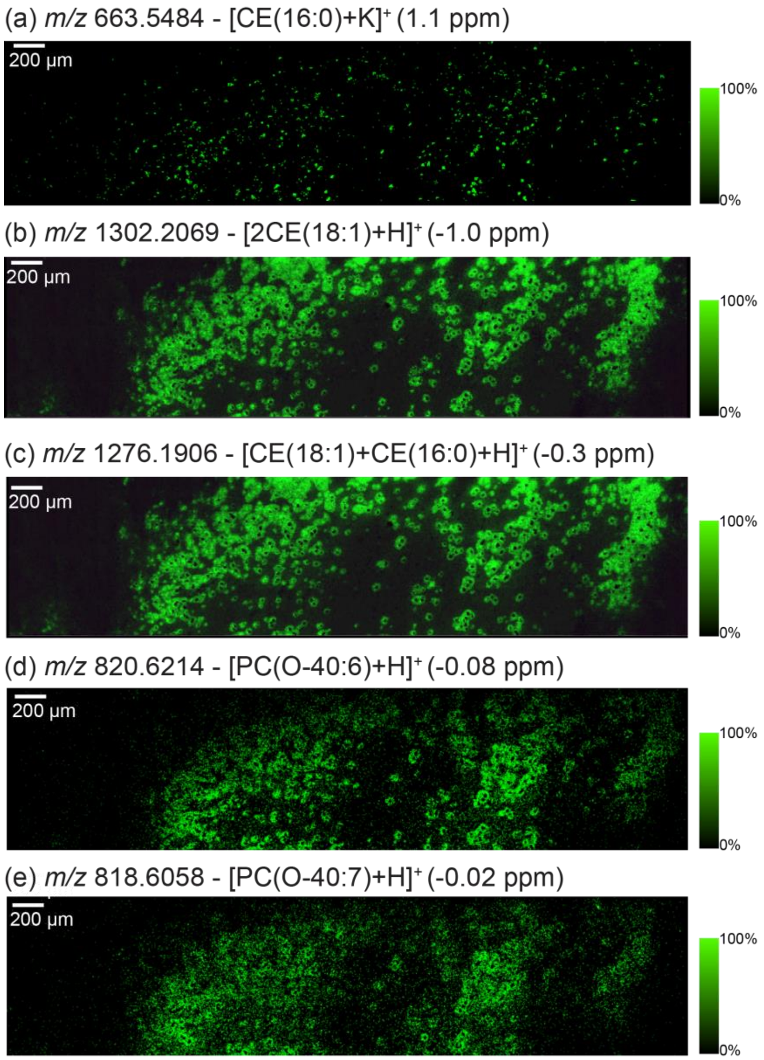

Fig. S10 MALDI-2-MSI ion distribution images acquired from human multiple sclerosis brain tissue using a 6 µm pixel size. (a) *m/z* 663.5484 ($[CE(16:0)+K]^+$, (b) *m/z* 1302.2069 ($[2CE(18:1)+H]^+$, (c) *m/z* 1276.1906 ($[CE(18:1)+CE(16:0)+H]^+$, (d) *m/z* 820.6214 ($[PC(O-40:6)+H]^+$ and (e) *m/z* 818.6058 ($[PC(O-40:7)+H]^+$. All images are visualized using total ion current normalization and hotspot removal 99% quantile

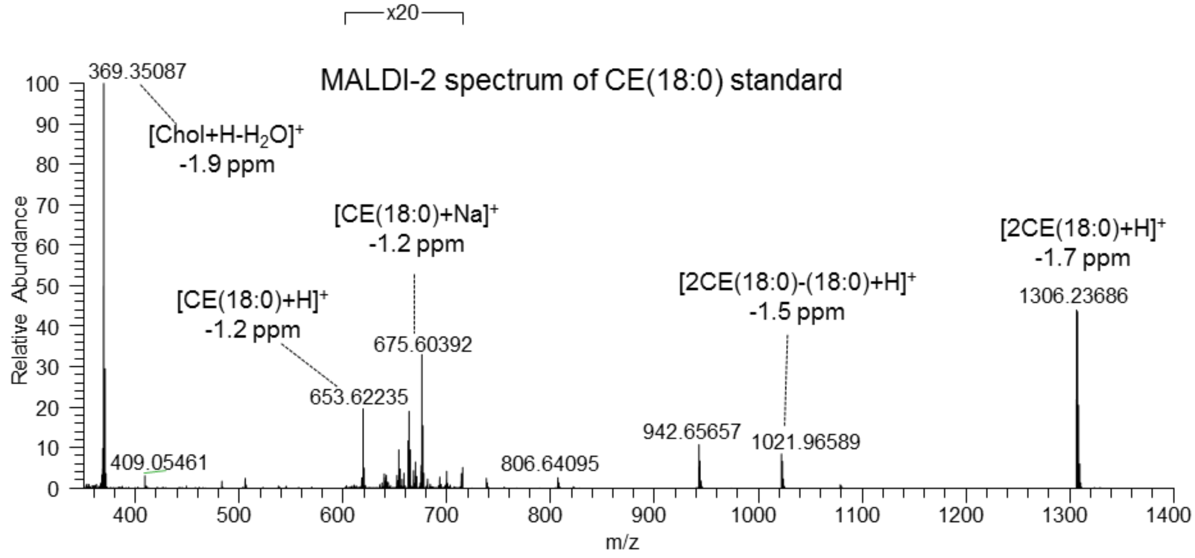

Fig. S11 MALDI-2 spectrum of a CE(18:0) standard demonstrating the formation of abundant $[2M+H]^+$ ions (*m/z* 1306.23686) as well as other CE(18:0)-related ions. A 1 µL aliquot of a 2 mM CE(18:0) standard was spotted onto an ITO slide. Once dried the ITO slide was sublimed with DHB and recrystallized using identical parameters as used to prepare the human brain tissue

References

1. . http://www.hmdb.ca/spectra/ei_ms/1143. Accessed 19-9-19

2. Ruiz-Jiménez J, Priego-Capote F, Mata-Granados JM, Quesada JM, Luque de Castro MD (2007) Determination of the ubiquinol-10 and ubiquinone-10 (coenzyme Q10) in human serum by liquid chromatography tandem mass spectrometry to evaluate the oxidative stress. Journal of Chromatography A 1175 (2):242-248.
