# Supplementary material for: Evaluation of lipid coverage and high spatial resolution MALDI-imaging capabilities of oversampling combined with laser post-ionisation
Source: Anal Bioanal Chem. 2019 Dec 26;412(10):2277–89. doi: 10.1007/s00216-019-02290-3 (PMC7118047; doi:10.1007/s00216-019-02290-3)
Supplement: Supplementary file 3 — (PDF 168 kb) [file 216_2019_2290_MOESM3_ESM.pdf]

Table S2. List of automatically identified lipid species from the mouse kidney tissue

Lipid class assignments are done so according to the nomenclature of the LIPIDMAPS database and showing only one match per *m/z*.

Notes:

1. For sterols many isomeric species are possible and thus identifications are assigned to the general "sterols" class.

2. 1-alkyl and 1-(1Z-alkenyl) chains cannot be distinguished. These lipids should be interpreted as belonging to a general ether sub-class (e.g., PC-O and PE-O) lipids. Note for 1-(1Z-alkenyl) chains the alkenyl double bond is not included in the number of unsaturated sites contained within the sum-composition formula. E.g., the plasmalogen PE(P-40:6) should instead be interpreted as PE(O-40:7).

3. For glycosphingolipids, the order and identity of the sugar groups is unknown and should just be interpreted as hexose (Hex) substituent. E.g., Hex2Cer(d42:1) instead of LacCer(d42:1).

4. In general identifications are tentative are based on accurate mass alone. The presence of isomeric and isobaric (within the achievable mass resolution) species cannot be excluded.

m/z	Formula	LIPIDMAPS Lipid Class	Sub-Class Abbreviation	Common Name	Neutral Mass	Mass delta ppm	Adduct	Normalized Intensity (0-100%)	Chaos score
367.3363	C27H44O	Sterols			384.3392	1	[M+H-H2O]+	6.1	98.9
369.3517	C27H46O	Sterols			386.3549	0.4	[M+H-H2O]+	32.7	98.48
502.2936	C25H44NO7P	Monoacylglycerophosphoethanolamines	PE	LPE(20:4)	501.2855	1.6	[M+H]+	0.2	77.83
526.2933	C27H44NO7P	Monoacylglycerophosphoethanolamines	PE	LPE(22:6)	525.2855	1	[M+H]+	0.2	84.32
689.5605	C38H77N2O6P	Ceramide phosphoethanolamines	PE-Cer	PE-Cer(d36:1)	688.5519	1.9	[M+H]+	0.1	74.33
701.5602	C39H77N2O6P	Ceramide phosphocholines (sphingomyelins)	SM	SM(d34:2)	700.5519	1.5	[M+H]+	0.1	71.02
702.5441	C39H76NO7P	1-alkyl,2-acylglycerophosphoethanolamines	PE	PE(O-34:2)	701.5359	1.2	[M+H]+	0.2	79.65
703.575	C39H79N2O6P	Ceramide phosphocholines (sphingomyelins)	SM	SM(d34:1)	702.5676	0.3	[M+H]+	1.3	96.09
716.5235	C39H74NO8P	Diacylglycerophosphoethanolamines	PE	PE(34:2)	715.5152	1.4	[M+H]+	0.6	93.34
718.5393	C39H76NO8P	Diacylglycerophosphoethanolamines	PE	PE(34:1)	717.5309	1.7	[M+H]+	0.5	90.88
720.555	C39H78NO8P	Diacylglycerophosphoethanolamines	PE	PE(34:0)	719.5465	1.7	[M+H]+	1.5	97.05
724.5287	C41H74NO7P	1-alkyl,2-acylglycerophosphoethanolamines	PE	PE(O-36:5)	723.5203	1.5	[M+H]+	5.0	97.98
726.5445	C41H76NO7P	1-alkyl,2-acylglycerophosphoethanolamines	PE	PE(O-36:4)	725.5359	1.8	[M+H]+	0.1	80.23
734.5702	C40H80NO8P	Diacylglycerophosphocholines	PC	PC(32:0)	733.5622	1.1	[M+H]+	7.2	98.47
738.5055	C41H72NO8P	Diacylglycerophosphoethanolamines	PE	PE(36:5)	737.4996	1.7	[M+H]+	0.2	78.47
738.5078	C41H72NO8P	Diacylglycerophosphoethanolamines	PE	PE(36:5)	737.4996	1.3	[M+H]+	0.5	92.07
740.5233	C41H74NO8P	Diacylglycerophosphoethanolamines	PE	PE(36:4)	739.5152	1.1	[M+H]+	10.3	98.65
742.539	C41H76NO8P	Diacylglycerophosphoethanolamines	PE	PE(36:3)	741.5309	1.2	[M+H]+	0.7	93.5

744.5552	C41H78NO8P	Diacylglycerophosphoethanolamines	PE	PE(36:2)	743.5465	1.8	[M+H]+	3.5	98.7
746.5707	C41H80NO8P	Diacylglycerophosphoethanolamines	PE	PE(36:1)	745.5622	1.8	[M+H]+	1.3	96.98
748.5283	C43H74NO7P	1-(1Z-alkenyl),2-acylglycerophosphoethanolamines	PE	PE(O-38:7)	747.5203	1	[M+H]+	2.5	98.17
749.5334	C40H77O10P	Diacylglycerophosphoglycerols	PG	PG(34:1)	748.5254	0.9	[M+H]+	0.4	93.45
750.5441	C43H76NO7P	1-alkyl,2-acylglycerophosphoethanolamines	PE	PE(O-38:6)	749.5359	1.2	[M+H]+	1.0	95.66
751.5474	C40H79O10P	Diacylglycerophosphoglycerols	PG	PG(34:0)	750.5411	1.3	[M+H]+	0.4	80.56
752.5597	C43H78NO7P	1-alkyl,2-acylglycerophosphoethanolamines	PE	PE(O-38:5)	751.5516	1.1	[M+H]+	3.9	98.21
754.5389	C42H76NO8P	Diacylglycerophosphocholines	PC	PC(34:4)	753.5309	1.1	[M+H]+	0.4	74.19
754.5757	C43H80NO7P	1-alkyl,2-acylglycerophosphoethanolamines	PE	PE(O-38:4)	753.5672	1.6	[M+H]+	0.2	85.74
756.5524	C42H78NO8P	Diacylglycerophosphocholines	PC	PC(34:3)	755.5465	1.9	[M+H]+	6.3	98.81
756.627	C44H86NO6P	Ceramide 1-phosphates	C1P	C1P(d44:2)	755.6193	0.5	[M+H]+	0.1	71.11
758.5706	C42H80NO8P	Diacylglycerophosphocholines	PC	PC(34:2)	757.5622	1.5	[M+H]+	3.9	98.17
760.5859	C42H82NO8P	Diacylglycerophosphocholines	PC	PC(34:1)	759.5778	1.1	[M+H]+	4.5	98.14
762.5056	C43H72NO8P	Diacylglycerophosphoethanolamines	PE	PE(38:7)	761.4996	1.6	[M+H]+	0.4	85.57
764.5232	C43H74NO8P	Diacylglycerophosphoethanolamines	PE	PE(38:6)	763.5152	0.9	[M+H]+	5.2	98.71
766.5389	C43H76NO8P	Diacylglycerophosphoethanolamines	PE	PE(38:5)	765.5309	1	[M+H]+	11.3	98.49
768.5538	C43H78NO8P	Diacylglycerophosphoethanolamines	PE	PE(38:4)	767.5465	0	[M+H]+	49.3	98.81
768.5901	C44H82NO7P	1-alkyl,2-acylglycerophosphocholines	PC	PC(O-36:4)	767.5829	0	[M+H]+	0.2	72.75
772.5855	C43H82NO8P	Diacylglycerophosphoethanolamines	PE	PE(38:2)	771.5778	0.6	[M+H]+	0.4	74.58
774.543	C45H76NO7P	1-(1Z-alkenyl),2-acylglycerophosphoethanolamines	PE	PE(O-40:8)	773.5359	0.3	[M+H]+	1.5	97.98
776.5601	C45H78NO7P	1-(1Z-alkenyl),2-acylglycerophosphoethanolamines	PE	PE(O-40:7)	775.5516	1.5	[M+H]+	1.5	98.06
778.5746	C45H80NO7P	1-alkyl,2-acylglycerophosphoethanolamines	PE	PE(O-40:6)	777.5672	0.1	[M+H]+	0.7	95.74
780.5527	C44H78NO8P	Diacylglycerophosphocholines	PC	PC(36:5)	779.5465	1.3	[M+H]+	3.3	98.68
780.59	C45H82NO7P	1-alkyl,2-acylglycerophosphoethanolamines	PE	PE(O-40:5)	779.5829	0.2	[M+H]+	0.1	89.1
782.5691	C44H80NO8P	Diacylglycerophosphocholines	PC	PC(36:4)	781.5622	0.5	[M+H]+	6.6	98.73
784.5852	C44H82NO8P	Diacylglycerophosphocholines	PC	PC(36:3)	783.5778	0.2	[M+H]+	0.7	85.77
786.6017	C44H84NO8P	Diacylglycerophosphocholines	PC	PC(36:2)	785.5935	1.2	[M+H]+	2.0	97.44
787.6698	C45H91N2O6P	Ceramide phosphocholines (sphingomyelins)	SM	SM(d40:1)	786.6615	1.3	[M+H]+	0.3	86.79
788.5215	C45H74NO8P	Diacylglycerophosphoethanolamines	PE	PE(40:8)	787.5152	1.3	[M+H]+	0.4	90.48
788.6177	C44H86NO8P	Diacylglycerophosphocholines	PC	PC(36:1)	787.6091	1.6	[M+H]+	0.6	94.47
790.5386	C45H76NO8P	Diacylglycerophosphoethanolamines	PE	PE(40:7)	789.5309	0.6	[M+H]+	3.4	96.21
790.5603	C42H80NO10P	Diacylglycerophosphoserines	PS	PS(36:1)	789.552	1.3	[M+H]+	0.2	76.13

790.5738	C46H80NO7P	1-(1Z-alkenyl),2-acylglycerophosphocholines	PC	PC(O-38:7)	789.5672	0.9	[M+H]+	0.3	79.26
792.5525	C45H78NO8P	Diacylglycerophosphoethanolamines	PE	PE(40:6)	791.5465	1.6	[M+H]+	0.2	97.52
792.5552	C45H78NO8P	Diacylglycerophosphoethanolamines	PE	PE(40:6)	791.5465	1.8	[M+H]+	4.4	98.41
792.5909	C46H82NO7P	1-alkyl,2-acylglycerophosphocholines	PC	PC(O-38:6)	791.5829	1	[M+H]+	3.7	96.21
794.5701	C45H80NO8P	Diacylglycerophosphoethanolamines	PE	PE(40:5)	793.5621	0.9	[M+H]+	0.7	92.21
796.586	C45H82NO8P	Diacylglycerophosphoethanolamines	PE	PE(40:4)	795.5778	1.1	[M+H]+	1.5	96.63
804.5525	C46H78NO8P	Diacylglycerophosphocholines	PC	PC(38:7)	803.5465	1.6	[M+H]+	2.8	98.44
806.5702	C46H80NO8P	Diacylglycerophosphocholines	PC	PC(38:6)	805.5622	1	[M+H]+	6.7	97.5
806.6067	C47H84NO7P	1-alkyl,2-acylglycerophosphoethanolamines	PE	PE(O-42:6)	805.5985	1.1	[M+H]+	0.2	77.49
808.5845	C46H82NO8P	Diacylglycerophosphocholines	PC	PC(38:5)	807.5778	0.7	[M+H]+	2.4	97.3
810.6022	C46H84NO8P	Diacylglycerophosphocholines	PC	PC(38:4)	809.5935	1.8	[M+H]+	2.3	97.13
812.5445	C44H78NO10P	Diacylglycerophosphoserines	PS	PS(38:4)	811.5363	1	[M+H]+	1.8	97.52
813.6851	C47H93N2O6P	Ceramide phosphocholines (sphingomyelins)	SM	SM(d42:2)	812.6771	0.9	[M+H]+	0.4	87.16
814.5368	C47H76NO8P	Diacylglycerophosphoethanolamines	PE	PE(42:9)	813.5309	1.6	[M+H]+	0.2	84.13
815.7008	C47H95N2O6P	Ceramide phosphocholines (sphingomyelins)	SM	SM(d42:1)	814.6928	0.9	[M+H]+	0.2	80.53
818.5691	C47H80NO8P	Diacylglycerophosphoethanolamines	PE	PE(42:7)	817.5622	0.5	[M+H]+	0.1	85.26
818.6056	C48H84NO7P	1-(1Z-alkenyl),2-acylglycerophosphocholines	PC	PC(O-40:7)	817.5985	0.3	[M+H]+	0.2	85.67
820.585	C47H82NO8P	Diacylglycerophosphoethanolamines	PE	PE(42:6)	819.5778	0.1	[M+H]+	0.4	89.47
830.5682	C48H80NO8P	Diacylglycerophosphocholines	PC	PC(40:8)	829.5622	1.5	[M+H]+	0.4	91.38
832.5835	C48H82NO8P	Diacylglycerophosphocholines	PC	PC(40:7)	831.5778	1.9	[M+H]+	2.3	97.32
834.6002	C48H84NO8P	Diacylglycerophosphocholines	PC	PC(40:6)	833.5935	0.7	[M+H]+	0.9	95.89
836.5447	C46H78NO10P	Diacylglycerophosphoserines	PS	PS(40:6)	835.5363	1.3	[M+H]+	0.2	73.94
856.5842	C50H82NO8P	Diacylglycerophosphocholines	PC	PC(42:9)	855.5778	1.1	[M+H]+	0.5	93.44
972.734	C54H101NO13	Simple Glc series	Hex2Cer	Hex2Cer(d42:2)	971.7273	0.6	[M+H]+	0.1	86.5
