# Supplementary material for: Evaluation of lipid coverage and high spatial resolution MALDI-imaging capabilities of oversampling combined with laser post-ionisation
Source: Anal Bioanal Chem. 2019 Dec 26;412(10):2277–89. doi: 10.1007/s00216-019-02290-3 (PMC7118047; doi:10.1007/s00216-019-02290-3)
Supplement: Supplementary file 4 — (PDF 246 kb) [file 216_2019_2290_MOESM4_ESM.pdf]

Table S3. List of automatically identified lipid species from the multiple sclerosis human brain tissue

Lipid class assignments are done so according to the nomenclature of the LIPIDMAPS database and showing only one match per *m/z*.

Notes:

1. For sterols many isomeric species are possible and thus identifications are assigned to the general "sterols" class.

2. 1-alkyl and 1-(1Z-alkenyl) chains cannot be distinguished. These lipids should be interpreted as belonging to a general ether sub-class (e.g., PC-O and PE-O) lipids. Note for 1-(1Z-alkenyl) chains the alkenyl double bond is not included in the number of unsaturated sites contained within the sum-composition formula. E.g., the plasmalogen PE(P-40:6) should instead be interpreted as PE(O-40:7).

3. For glycosphingolipids, the order and identity of the sugar groups is unknown and should just be interpreted as hexose (Hex) substituents. E.g., Hex2Cer(d42:1) instead of LacCer(d42:1).

4. In general identifications are tentative are based on accurate mass alone. The presence of isomeric and isobaric (within the achievable mass resolution) species cannot be excluded.

m/z	Formula	LIPIDMAPS Lipid Class	Sub-Class Abbreviation	Common Name	Mass delta ppm	Adduct	Normalized Intensity (0-100%)[a]	Chaos score
361.2743	C23H38O4	Sterols			1.5	[M+H-H2O]+	1.1	97.45
367.3361	C27H44O	Sterols			0.3	[M+H-H2O]+	23.1	99.29
369.3517	C27H46O	Sterols			0.3	[M+H-H2O]+	100.0	99.2
371.3313	C26H44O2	Sterols			1.1	[M+H-H2O]+	0.2	83.23
381.3158	C27H42O2	Sterols			1.6	[M+H-H2O]+	0.2	72.2
383.3313	C27H44O2	Sterols			1.3	[M+H-H2O]+	3.3	96.83
385.2743	C25H38O4	Sterols			1.4	[M+H-H2O]+	0.7	96.43
389.3056	C25H42O4	Sterols			1.4	[M+H-H2O]+	0.5	96.1
401.3419	C27H46O3	Sterols			1.1	[M+H-H2O]+	0.3	81.29
454.2933	C21H44NO7P	Monoacylglycerophosphoethanolamines	LPE	LPE(16:0)	1	[M+H]+	0.1	78.6
480.3088	C23H46NO7P	Monoacylglycerophosphoethanolamines	LPE	LPE(18:1)	0.7	[M+H]+	1.9	98.58
482.3246	C23H48NO7P	Monoalkylglycerophosphoethanolamines	LPE	LPE(18:0)	0.9	[M+H]+	0.2	72.67
502.2933	C25H44NO7P	Monoacylglycerophosphoethanolamines	LPE	LPE(20:4)	1	[M+H]+	2.1	98.69
508.3402	C25H50NO7P	Monoacylglycerophosphoethanolamines	LPE	LPE(20:1)	0.9	[M+H]+	0.4	97.73
526.2932	C27H44NO7P	Monoacylglycerophosphoethanolamines	LPE	LPE(22:6)	0.8	[M+H]+	1.5	98.21
530.3245	C27H48NO7P	Monoacylglycerophosphoethanolamines	LPE	LPE(22:4)	0.7	[M+H]+	0.7	97.85
566.551	C36H71NO3	N-acylsphingosines (ceramides)	Cer	Cer(d36:1)	0.6	[M+H]+	1.9	98.33
648.6292	C42H81NO3	N-acylsphingosines (ceramides)	Cer	Cer(d42:2)	0.5	[M+H]+	3.6	99.19
650.6452	C42H83NO3	N-acylsphinganines (dihydroceramides)	Cer	Cer(d42:1)	0.9	[M+H]+	0.4	95.45

663.5484	C43H76O2	Steryl esters	CE	CE(16:0)	1	[M+K]+	0.1	73.81
664.6242	C42H81NO4	N-acylsphingosines (ceramides)	Cer	Cer(d42:2)	0.6	[M+H]+	0.4	97.94
689.5603	C38H77N2O6P	Ceramide phosphoethanolamines	PE-Cer	PE-Cer(d36:1)	1.5	[M+H]+	0.8	96.2
689.5624	C45H78O2	Steryl esters	CE	CE(18:1)	1.3	[M+K]+	0.6	74.05
700.5278	C39H74NO7P	1-alkyl,2-acylglycerophosphoethanolamines	PE	PE(O-34:3)	0.4	[M+H]+	0.1	72.76
702.5435	C39H76NO7P	1-alkyl,2-acylglycerophosphoethanolamines	PE	PE(O-34:2)	0.4	[M+H]+	12.0	99.2
703.5752	C39H79N2O6P	Ceramide phosphocholines (sphingomyelins)	SM	SM(d34:1)	0.6	[M+H]+	1.7	96.87
704.5594	C39H78NO7P	1-alkyl,2-acylglycerophosphoethanolamines	PE	PE(O-34:1)	0.7	[M+H]+	0.8	95.98
706.5385	C38H76NO8P	Diacylglycerophosphocholines	PC	PC(30:0)	0.5	[M+H]+	0.7	93.24
714.5435	C40H76NO7P	1-alkyl,2-acylglycerophosphocholines	PC	PC(O-32:3)	0.4	[M+H]+	0.2	90.36
716.5228	C39H74NO8P	Diacylglycerophosphoethanolamines	PE	PE(34:2)	0.5	[M+H]+	0.8	97.58
716.5592	C40H78NO7P	1-alkyl,2-acylglycerophosphocholines	PC	PC(O-32:2)	0.4	[M+H]+	0.5	97.86
717.5909	C40H81N2O6P	Ceramide phosphoethanolamines	PE-Cer	PE-Cer(d38:1)	0.5	[M+H]+	2.0	97.89
718.5384	C39H76NO8P	Diacylglycerophosphoethanolamines	PE	PE(34:1)	0.4	[M+H]+	10.9	99.16
720.5544	C39H78NO8P	Diacylglycerophosphoethanolamines	PE	PE(34:0)	0.9	[M+H]+	1.7	98.04
724.5279	C41H74NO7P	1-alkyl,2-acylglycerophosphoethanolamines	PE	PE(O-36:5)	0.4	[M+H]+	3.4	94.82
726.5436	C41H76NO7P	1-alkyl,2-acylglycerophosphoethanolamines	PE	PE(O-36:4)	0.6	[M+H]+	2.9	98.94
726.5882	C42H79NO8	Simple Glc series	HexCer	HexCer(d36:2)	0.5	[M+H]+	1.3	98.81
728.5593	C41H78NO7P	1-alkyl,2-acylglycerophosphoethanolamines	PE	PE(O-36:3)	0.6	[M+H]+	14.2	98.89
728.6038	C42H81NO8	Simple Glc series	HexCer	HexCer(d36:1)	0.4	[M+H]+	10.2	99.22
729.591	C41H81N2O6P	Ceramide phosphocholines (sphingomyelins)	SM	SM(d36:2)	0.7	[M+H]+	0.6	95.11
730.5384	C40H76NO8P	Diacylglycerophosphocholines	PC	PC(32:2)	0.4	[M+H]+	0.3	73.65
730.5752	C41H80NO7P	1-alkyl,2-acylglycerophosphoethanolamines	PE	PE(O-36:2)	1	[M+H]+	5.3	98.69
731.6064	C41H83N2O6P	Ceramide phosphocholines (sphingomyelins)	SM	SM(d36:1)	0.4	[M+H]+	18.6	98.52
732.5542	C40H78NO8P	Diacylglycerophosphocholines	PC	PC(32:1)	0.5	[M+H]+	4.2	98.59
732.5908	C41H82NO7P	1-alkyl,2-acylglycerophosphoethanolamines	PE	PE(O-36:1)	0.9	[M+H]+	0.2	89.77
734.5698	C40H80NO8P	Diacylglycerophosphocholines	PC	PC(32:0)	0.6	[M+H]+	12.1	98.43
738.5433	C42H76NO7P	1-(1Z-alkenyl),2-acylglycerophosphocholines	PC	PC(O-34:5)	0.1	[M+H]+	0.3	92.71
740.5227	C41H74NO8P	Diacylglycerophosphoethanolamines	PE	PE(36:4)	0.2	[M+H]+	1.4	98.52
742.5376	C41H76NO8P	Diacylglycerophosphoethanolamines	PE	PE(36:3)	0.7	[M+H]+	0.9	97.73
742.5748	C42H80NO7P	1-alkyl,2-acylglycerophosphocholines	PC	PC(O-34:3)	0.4	[M+H]+	0.2	94.06
744.5541	C41H78NO8P	Diacylglycerophosphoethanolamines	PE	PE(36:2)	0.4	[M+H]+	15.6	99.21
744.5984	C42H81NO9	Simple Glc series	HexCer	HexCer(d36:1)	0	[M+H]+	0.3	97.64

745.6222	C42H85N2O6P	Ceramide phosphoethanolamines	PE-Cer	PE-Cer(d40:1)	0.6	[M+H]+	0.2	76.67
746.57	C41H80NO8P	Diacylglycerophosphoethanolamines	PE	PE(36:1)	0.8	[M+H]+	16.1	99.03
746.606	C42H84NO7P	1-alkyl,2-acylglycerophosphocholines	PC	PC(O-34:1)	0.2	[M+H]+	0.6	97.82
748.5277	C43H74NO7P	1-(1Z-alkenyl),2-acylglycerophosphoethanolamines	PE	PE(O-38:7)	0.1	[M+H]+	3.1	98.82
748.5857	C41H82NO8P	Diacylglycerophosphoethanolamines	PE	PE(36:0)	0.8	[M+H]+	0.4	86.04
749.532	C40H77O10P	Diacylglycerophosphoglycerols	PG	PG(34:1)	0.9	[M+H]+	0.8	97.82
750.5434	C43H76NO7P	1-alkyl,2-acylglycerophosphoethanolamines	PE	PE(O-38:6)	0.2	[M+H]+	17.8	99.12
752.5594	C43H78NO7P	1-alkyl,2-acylglycerophosphoethanolamines	PE	PE(O-38:5)	0.7	[M+H]+	11.6	99.05
754.5746	C43H80NO7P	1-alkyl,2-acylglycerophosphoethanolamines	PE	PE(O-38:4)	0.1	[M+H]+	2.3	98.86
756.5906	C43H82NO7P	1-alkyl,2-acylglycerophosphoethanolamines	PE	PE(O-38:3)	0.6	[M+H]+	3.1	98.73
756.6349	C44H85NO8	Simple Glc series	HexCer	HexCer(d38:1)	0.2	[M+H]+	0.1	84.36
758.5696	C42H80NO8P	Diacylglycerophosphocholines	PC	PC(34:2)	0.2	[M+H]+	1.3	98.16
758.6064	C43H84NO7P	1-alkyl,2-acylglycerophosphoethanolamines	PE	PE(O-38:2)	0.7	[M+H]+	1.4	98.71
759.6377	C43H87N2O6P	Ceramide phosphocholines (sphingomyelins)	SM	SM(d38:1)	0.4	[M+H]+	0.9	97.1
760.5852	C42H82NO8P	Diacylglycerophosphocholines	PC	PC(34:1)	0.1	[M+H]+	51.7	98.67
762.5068	C43H72NO8P	Diacylglycerophosphoethanolamines	PE	PE(38:7)	0.1	[M+H]+	0.2	76.3
762.6017	C42H84NO8P	Diacylglycerophosphocholines	PC	PC(34:0)	1.3	[M+H]+	0.4	90.69
763.5471	C41H79O10P	Diacylglycerophosphoglycerols	PG	PG(34:0)	1.6	[M+H]+	0.1	82.71
764.5226	C43H74NO8P	Diacylglycerophosphoethanolamines	PE	PE(38:6)	0.1	[M+H]+	6.5	99.15
764.5589	C44H78NO7P	1-(1Z-alkenyl),2-acylglycerophosphocholines	PC	PC(O-36:6)	0	[M+H]+	0.6	92.51
766.5383	C43H76NO8P	Diacylglycerophosphoethanolamines	PE	PE(38:5)	0.3	[M+H]+	14.5	99.19
766.5745	C44H80NO7P	1-alkyl,2-acylglycerophosphocholines	PC	PC(O-36:5)	0	[M+H]+	0.4	94.6
768.554	C43H78NO8P	Diacylglycerophosphoethanolamines	PE	PE(38:4)	0.3	[M+H]+	28.1	99.13
768.5892	C44H82NO7P	1-alkyl,2-acylglycerophosphocholines	PC	PC(O-36:4)	1.2	[M+H]+	0.2	95.44
770.5691	C43H80NO8P	Diacylglycerophosphoethanolamines	PE	PE(38:3)	0.5	[M+H]+	0.3	89.18
770.6059	C44H84NO7P	1-alkyl,2-acylglycerophosphocholines	PC	PC(O-36:3)	0.1	[M+H]+	0.1	85.82
772.5851	C43H82NO8P	Diacylglycerophosphoethanolamines	PE	PE(38:2)	0	[M+H]+	1.3	98.05
774.5432	C45H76NO7P	1-(1Z-alkenyl),2-acylglycerophosphoethanolamines	PE	PE(O-40:8)	0	[M+H]+	2.8	97.78
774.6009	C43H84NO8P	Diacylglycerophosphoethanolamines	PE	PE(38:1)	0.3	[M+H]+	3.6	98.83
776.559	C45H78NO7P	1-(1Z-alkenyl),2-acylglycerophosphoethanolamines	PE	PE(O-40:7)	0.2	[M+H]+	13.9	97.92
778.5376	C44H76NO8P	Diacylglycerophosphocholines	PC	PC(36:6)	0.7	[M+H]+	0.3	93.03
778.5748	C45H80NO7P	1-alkyl,2-acylglycerophosphoethanolamines	PE	PE(O-40:6)	0.4	[M+H]+	3.7	98
780.5527	C44H78NO8P	Diacylglycerophosphocholines	PC	PC(36:5)	1.4	[M+H]+	1.1	98.36

780.5906	C45H82NO7P	1-alkyl,2-acylglycerophosphoethanolamines	PE	PE(O-40:5)	0.5	[M+H]+	8.3	98.32
782.5331	C43H76NO9P	Oxidized glycerophosphocholines	PE	PE(38:4(KE))	0.1	[M+H]+	0.2	84.58
782.6065	C45H84NO7P	1-alkyl,2-acylglycerophosphoethanolamines	PE	PE(O-40:4)	0.9	[M+H]+	0.6	97.23
782.6505	C46H87NO8	Simple Glc series	HexCer	HexCer(d40:2)	0	[M+H]+	2.0	98.9
784.5847	C44H82NO8P	Diacylglycerophosphocholines	PC	PC(36:3)	0.5	[M+H]+	0.5	94.36
784.6215	C45H86NO7P	1-alkyl,2-acylglycerophosphoethanolamines	PE	PE(O-40:3)	0.1	[M+H]+	0.5	97.61
784.6663	C46H89NO8	Simple Glc series	HexCer	HexCer(d40:1)	0.2	[M+H]+	0.6	98.39
786.601	C44H84NO8P	Diacylglycerophosphocholines	PC	PC(36:2)	0.3	[M+H]+	3.4	98.4
786.6374	C45H88NO7P	1-alkyl,2-acylglycerophosphoethanolamines	PE	PE(O-40:2)	0.3	[M+H]+	0.1	80.61
788.5211	C45H74NO8P	Diacylglycerophosphoethanolamines	PE	PE(40:8)	1.7	[M+H]+	0.2	89.05
788.5238	C45H74NO8P	Diacylglycerophosphoethanolamines	PE	PE(40:8)	1.7	[M+H]+	0.0	71.81
788.5439	C42H78NO10P	Diacylglycerophosphoserines	PS	PS(36:2)	0.3	[M+H]+	1.0	97.49
788.6166	C44H86NO8P	Diacylglycerophosphocholines	PC	PC(36:1)	0.3	[M+H]+	8.9	98.63
790.538	C45H76NO8P	Diacylglycerophosphoethanolamines	PE	PE(40:7)	0.2	[M+H]+	5.0	99.19
790.5595	C42H80NO10P	Diacylglycerophosphoserines	PS	PS(36:1)	0.3	[M+H]+	9.9	99
792.5538	C45H78NO8P	Diacylglycerophosphoethanolamines	PE	PE(40:6)	0	[M+H]+	37.2	99.24
794.5695	C45H80NO8P	Diacylglycerophosphoethanolamines	PE	PE(40:5)	0.1	[M+H]+	1.0	96.9
794.6058	C46H84NO7P	1-alkyl,2-acylglycerophosphocholines	PC	PC(O-38:5)	0.1	[M+H]+	0.1	74.67
796.5853	C45H82NO8P	Diacylglycerophosphoethanolamines	PE	PE(40:4)	0.2	[M+H]+	7.3	98.83
800.661	C46H89NO9	Simple Glc series	HexCer	HexCer(d40:1)	0	[M+H]+	3.8	99.16
804.5527	C46H78NO8P	Diacylglycerophosphocholines	PC	PC(38:7)	1.4	[M+H]+	0.9	98.61
806.5692	C46H80NO8P	Diacylglycerophosphocholines	PC	PC(38:6)	0.3	[M+H]+	0.9	98.11
806.6059	C47H84NO7P	1-alkyl,2-acylglycerophosphoethanolamines	PE	PE(O-42:6)	0	[M+H]+	0.8	98.25
807.6094	C44H87O10P	Diacylglycerophosphoglycerols	PG	PG(38:0)	2	[M+H]+	0.2	94.99
808.5489	C45H78NO9P	Oxidized glycerophosphoethanolamines	PE	PE(40:6(OH))	0.2	[M+H]+	0.3	96.38
808.5837	C46H82NO8P	Diacylglycerophosphocholines	PC	PC(38:5)	1.7	[M+H]+	1.2	98.55
808.6217	C47H86NO7P	1-(1Z-alkenyl),2-acylglycerophosphoethanolamines	PE	PE(O-42:5)	0.3	[M+H]+	0.4	97.96
810.5997	C46H84NO8P	Diacylglycerophosphocholines	PC	PC(38:4)	1.3	[M+H]+	2.4	98.78
810.6819	C48H91NO8	Simple Glc series	HexCer	HexCer(d42:2)	0.2	[M+H]+	31.5	99.2
812.5429	C44H78NO10P	Diacylglycerophosphoserines	PS	PS(38:4)	0.9	[M+H]+	0.5	95.1
812.6989	C48H93NO8	Simple Glc series	HexCer	HexCer(d42:1)	1.8	[M+H]+	4.4	97.63
813.6844	C47H93N2O6P	Ceramide phosphocholines (sphingomyelins)	SM	SM(d42:2)	0	[M+H]+	1.4	97.97
814.5592	C44H80NO10P	Diacylglycerophosphoserines	PS	PS(38:3)	0.1	[M+H]+	0.3	84.14
814.714	C48H95NO8	Simple Glc series	HexCer	HexCer(d42:0)	1.1	[M+H]+	0.1	71.75

816.575	C44H82NO10P	Diacylglycerophosphoserines	PS	PS(38:2)	0.1	[M+H]+	0.5	92.2
816.6831	C47H94NO7P	1-alkyl,2-acylglycerophosphoethanolamines	PE	PE(O-42:1)	1.2	[M+H]+	0.1	70.39
818.5691	C47H80NO8P	Diacylglycerophosphoethanolamines	PE	PE(42:7)	0.3	[M+H]+	0.1	74.62
818.5907	C44H84NO10P	Diacylglycerophosphoserines	PS	PS(38:1)	0.2	[M+H]+	0.5	97.96
818.6058	C48H84NO7P	1-(1Z-alkenyl),2-acylglycerophosphocholines	PC	PC(O-40:7)	0	[M+H]+	0.2	93.46
820.6214	C48H86NO7P	1-alkyl,2-acylglycerophosphocholines	PC	PC(O-40:6)	0	[M+H]+	0.3	95.06
826.6768	C48H91NO9	Simple Glc series	HexCer	HexCer(d42:2)	0.2	[M+H]+	16.3	99.21
828.6927	C48H93NO9	Simple Glc series	HexCer	HexCer(d42:1)	0.5	[M+H]+	19.4	99.26
830.7013	C48H96NO7P	1-alkyl,2-acylglycerophosphocholines	PC	PC(O-40:1)	1.9	[M+H]+	0.8	93.08
834.6003	C48H84NO8P	Diacylglycerophosphocholines	PC	PC(40:6)	0.5	[M+H]+	0.4	93.67
836.5434	C46H78NO10P	Diacylglycerophosphoserines	PS	PS(40:6)	0.2	[M+H]+	2.7	98.71
838.5584	C46H80NO10P	Diacylglycerophosphoserines	PS	PS(40:5)	1.1	[M+H]+	0.2	83.3
838.7132	C50H95NO8	Simple Glc series	HexCer	HexCer(d44:2)	0.2	[M+H]+	5.0	99.16
840.5746	C46H82NO10P	Diacylglycerophosphoserines	PS	PS(40:4)	0.4	[M+H]+	0.4	88.36
840.7283	C50H97NO8	Simple Glc series	HexCer	HexCer(d44:1)	0.5	[M+H]+	0.2	79.18
848.6377	C46H90NO10P	Diacylglycerophosphoserines	PS	PS(40:0)	0.2	[M+H]+	0.6	96.71
868.6066	C48H86NO10P	Diacylglycerophosphoserines	PS	PS(42:4)	0.5	[M+H]+	0.2	96.42
884.542	C50H78NO10P	Diacylglycerophosphoserines	PS	PS(44:10)	1.8	[M+H]+	0.1	71.29
886.5579	C50H80NO10P	Diacylglycerophosphoserines	PS	PS(44:9)	1.5	[M+H]+	0.2	90.39
887.564	C47H83O13P	Diacylglycerophosphoinositols	PI	PI(38:4)	0.5	[M+H]+	0.6	96.26
888.5736	C50H82NO10P	Diacylglycerophosphoserines	PS	PS(44:8)	1.4	[M+H]+	0.2	90.13
890.6566	C48H91NO13	Gal- (Gala series)	Hex2Cer	Hex2Cer(d36:1)	0.3	[M+H]+	0.2	72.46
972.7347	C54H101NO13	Gal- (Gala series)	Hex2Cer	Hex2Cer(d42:2)	0.1	[M+H]+	0.3	94.19
974.7507	C54H103NO13	Gal- (Gala series)	Hex2Cer	Hex2Cer(d42:1)	0.5	[M+H]+	0.1	74.44
1052.7085	C54H101NO18	Galα1-4Galβ1-4Glc- (Globo series)	Hex3Cer	Hex3Cer(d36:1)	0.7	[M+H]+	0.1	72.46
1255.7885	C62H114N2O23	GalNAcβ1-4Galβ1-4Glc- (Ganglio series)	GalNAcβ1-3Galα1-3Galβ1-4Glcβ-Cer	GM1(d36:1)	0	[M+H]+	0.3	92.69
1283.8194	C64H118N2O23	GalNAcβ1-4Galβ1-4Glc- (Ganglio series)	GalNAcβ1-3Galα1-3Galβ1-4Glcβ-Cer	GM1(d38:1)	0.3	[M+H]+	0.3	90.29
1337.8657	C68H124N2O23	GalNAcβ1-4Galβ1-4Glc- (Ganglio series)	GalNAcβ1-3Galα1-3Galβ1-4Glcβ-Cer	GM1(d42:2)	0.8	[M+H]+	0.1	78.3

a base peak corresponds to the radical cation of Coenzyme Q9 at *m/z* 794.6211. As this was not automatically identified it is not included in the list..
